# Tumor-associated macrophage-derived exosomes LINC01592 induce the immune escape of esophageal cancer by decreasing MHC-I surface expression

**DOI:** 10.1186/s13046-023-02871-2

**Published:** 2023-11-02

**Authors:** Xinwei Qiao, Zaixing Cheng, Kaming Xue, Cui Xiong, Zhikun Zheng, Xin Jin, Jinsong Li

**Affiliations:** 1 Department of Thoracic Surgery, Union Hospital, Tongji Medical College, Huazhong University of Science and Technology, 1277 Jiefang Avenue, Wuhan, 430022 Hubei China; 2 Department of Traditional Chinese Medicine, Union Hospital, Tongji Medical College, Huazhong University of Science and Technology, 1277 Jiefang Avenue, Wuhan, 430022 Hubei China; 3 Department of Endocrinology, Union Hospital, Tongji Medical College, Huazhong University of Science and Technology, 1277 Jiefang Avenue, Wuhan, 430022 Hubei China; 4 Department of Urology, The Second Xiangya Hospital, Central South University, Changsha, 410011 Hunan China

**Keywords:** TAMs, Esophageal cancer, Immune Escape, LINC01592/E2F6/NBR1/MHC-I, CD8^+^ CTL

## Abstract

**Background:**

TAMs (tumor-associated macrophages) infiltration promotes the progression of esophageal cancer (EC). However, the underlying mechanisms remain unclear.

**Methods:**

Abnormal expression of LINC01592 from EC microarrays of the TCGA database was analyzed. LINC01592 expression level was validated in both EC cell lines and tissues. Stable LINC01592 knockdown and overexpression of EC cell lines were established. In vitro and in vivo trials were conducted to test the impact of LINC01592 knockdown and overexpression on EC cells. RNA binding protein immunoprecipitation (RIP), RNA pulldown assays, and Immunofluorescence (IF) were used to verify the combination of E2F6 and LINC01592. The combination of E2F6 and NBR1 was verified through the utilization of ChIP and dual luciferase reporter assays.

**Results:**

LINC01592 is carried and transferred by exosomes secreted by M2-TAMs to tumor cells. The molecular mechanism underlying the promotion of NBR1 transcription involves the direct binding of LINC01592 to E2F6, which facilitates the nuclear entry of E2F6. The collaborative action of LINC01592 and E2F6 results in improved NBR1 transcription. The elevation of NBR1 binding to the ubiquitinated protein MHC-I via the ubiquitin domain caused a higher degradation of MHC-I in autophagolysosomes and a reduction in MHC-I expression on the exterior of cancerous cell. Consequently, this caused cancerous cells to escape from CD8^+^ CTL immune attack. The tumor-promoting impacts of LINC01592, as well as the growth of M2-type macrophage-driven tumors, were significantly suppressed by the interruption of E2F6/NBR1/MHC-I signaling through the effect of siRNA or the corresponding antibody blockade. Significantly, the suppression of LINC01592 resulted in an upregulation of MHC-I expression on the tumor cell membrane, thereby enhancing the efficacy of CD8+ T cell reinfusion therapy.

**Conclusions:**

The investigation conducted has revealed a significant molecular interaction between TAMs and EC via the LINC01592/E2F6/NBR1/MHC-I axis, which facilitates the progression of malignant tumors. This suggests that a therapeutic intervention targeting this axis may hold promise for the treatment of the disease.

**Supplementary Information:**

The online version contains supplementary material available at 10.1186/s13046-023-02871-2.

## Background

Esophageal cancer (EC) is the sixth most widespread malignancy globally in terms of mortality and the most aggressive and lethal gastrointestinal cancer that resists conventional therapies [1, 2]. TME (the tumor microenvironment) is a critical factor in facilitating the malignant proliferation and advancement of EC [3, 4]. Tumor-associated macrophages (TAMs) are a significant subset of immune cells that infiltrate tumors and are pivotal in mediating the interactions between the immune system and malignant cells. TAMs represent a prevalent type of immune cells within the context of solid tumors [5, 6]. In most human cancers, including EC, the infiltration of TAMs and the upregulation of associated gene expression seriously affect the prognosis and therapeutic effect of tumors [5]. It is still unknown how TAMs have a vital function in the malignancy of ECs.

Exosomes have a wide variety of functional mRNAs, miRNAs, and proteins. These exosomes are known to facilitate intercellular communication between immune and cancerous cells within the microenvironment of the tumor by transferring these genetic materials and proteins. The involvement of exosomes originating from cancerous cells in the progression of cancer is facilitated by intercellular communication between cancer and adjacent stromal tissue, activation of mechanisms that promote growth and angiogenesis, initiation of the pre-metastatic microenvironment formation, and inhibition of immune responses [7]. Investigations have discovered that TAMs can transport miRNAs to cancer cell, secrete exosome, and promote tumor cells to develop drug resistance. Exosomes originating from TAMs have been found to decrease the responsiveness of pancreatic ductal adenocarcinoma to gemcitabine both in vivo and in vitro. This effect is achieved through the transfer of miR-365 [8]. In recent years, an increasing number of investigations have revealed that exosomes originating from TAMs possess the ability to enhance tumor immune escape by suppressing immunity. The influence of tumor-derived exosomes on the cell cycle and cell migration of human EC cell lines was explored by Matsumoto Y et al. [9]. The investigation conducted by Jin Y et al. revealed that the progression and development of esophageal squamous cell carcinoma are facilitated by exosomal miR-3656 originated from cancer-related fibroblasts through the ACAP2/PI3K-AKT signaling mechanism [10]. Zhao Q et al. have reported the induction of the generation of monocytic myeloid-derived suppressor cells by fibroblasts linked to tumors. This process promotes resistance to cisplatin in EC [11]. Li W et al. found that the exosomal FMR1-AS1 molecule has a function in preserving the dynamic equilibrium of cancer stem-like cells in female esophageal carcinoma, which is achieved through the triggering of TLR7/NF-κB/c-Myc signaling mechanism [12]. Evidence suggests that exosomes are important for tumor progression.

The present investigation employed M0/M2-TAM exosomes that had been previously isolated for high-throughput sequencing to investigate the function of TAM exosomes in the immune evasion of EC. The study revealed a significant upregulation of LINC01592 expression in exosomes derived from M2-TAM, and the functional assays validated that LINC01592-enriched exosomes facilitated immune evasion of EC cells by avoiding CD8^+^ CTL cells. Additionally, the prevention of M2-TAMs from evading the immune system can be achieved by suppressing the formation of exosomes or reducing the expression of LINC01592. A significant factor contributing to the immune evasion of tumors is the insufficient presentation of MHC class I molecules [13]. By promoting MHC-I antigen processing pathway cooperative silencing, polycomb repressive complex 2 (PRC2) promotes T cell-mediated immune escape, for example [14]. According to Yamamoto K et al., NBR1 interacts with MHC-I via its ubiquitin-binding domain, mediates MHC-I degradation in autophagolysosomes, and provides a mechanism for activating autophagy, and therefore promotes immune escape [15]. In our investigation, we discovered that LINC01592 originated from M2-TAM exosome and advanced EC immune escape via the E2F6/NBR1/MHC-I axis. The siRNA or antibody blockade of LINC01592/E2F6/NBR1/MHC-I greatly diminished LINC01592's tumor-supporting effects and suppressed M2-dependent tumor growth.

## Materials and methods

### Clinical samples

The EC samples were surgically obtained from Wuhan Union Hospital. Tables S1-2 contain an illustration of the patient's features. Informed consent forms were signed by all patients prior to specimen collection. In this study, the Declaration of Helsinki guidelines were followed, and all relevant ethical approvals were acquired. This study has been approved by the Institutional Review Board (IRB, S682). Additional information is presented in the supplementary materials.

### Cell culture and treatment

For cell culture, we utilized the technique defined in our prior publications [16–18]. Supplemental materials provide detailed methods.

### Plasmids, small interfering RNA (siRNA), and transfection

The siRNAs utilized in this study were acquired from GeneChem Co. Ltd. (Shanghai, China), and Table S3 lists them. Following the manufacturer's instructions, all protocols were followed. Details on the approaches can be found in the supplementary material.

### Western blotting (WB)

The associated protocol information has been published in our prior investigations [16, 17, 19, 20]. Table S4 contains a comprehensive list of all antibodies and their corresponding details.

### Real-time quantitative RT-PCR (qRT-PCR)

Following the manufacturer's instructions, this assay was performed. Normalizing the data to GAPDH, which functions as a control, was achieved using the 2^–ΔΔCt^ method [21]. This study used primers synthesized by GeneCreate (Wuhan, China). Table S5 lists primer sequences, and supplementary materials provide more details.

### Bioinformatic analysis

Data were collected from the Genotype-Tissue Expression (GTEx) and TCGA databases (https://cancergenome.nih.gov/) [22]. A comprehensive explanation of the methodology is available in the supplementary materials.

### Isolation and identification of exosomes

Ultracentrifugation was used to purify exosomes according to standard operating procedures. Protocols are detailed in previous articles [23–25].

### Chromatin immunoprecipitation (ChIP)

The ChIP assay kits utilized in the study were produced by Upstate Biotechnology, located in Temecula, California. Sonication was conducted after formaldehyde crosslinking. Protein A/G beads were pretreated before incubation with anti-E2F6 antibodies. IgG was employed as a negative control in the experiment. Using QIAGEN DNA extraction kits, DNA was extracted from complexes, and qRT-PCR was conducted. Table S6 lists the primers utilized in ChIP-qPCR.

### Dual luciferase reporter assays

A dual-luciferase reporter assay was conducted employing the manufacturer's rules (Promega). Briefly, a luciferase reporter was built by utilizing complementary oligonucleotides that encompassed potential NBR1 binding locations (both types; wild (wt) and mutant (mut) and introducing them into pGL3-control luciferase reporter vectors. The protocol is detailed in our previous articles [16, 26, 27].

### Subcellular fractionation analysis

In this study, RNA subcellular fractionation was performed employing the kit of PARISTM (Ambion, Austin, TX). In order to detect the RNA content in the nucleus and cytoplasm, qRT-PCR was used. The internal controls for cytoplasm and nucleus were utilized, specifically β-actin and U6, respectively. The Minute™ Cytoplasmic and Nuclear Extraction Kit (Invent, cat#SC-003) was utilized to obtain proteins. Tubulin and lamin B1 functioned as internal references for the nucleus and cytoplasm, respectively.

### FISH, IF and IHC

A FISH assay was performed using detection probes from LINC01592 (RIBOBIO) and Bosterbio FISH kits. We performed FISH, IF, and IHC tests and scored them as previously described [16, 23, 28, 29]. This experiment used the antibodies listed in Table S4.

### Co-IP (Coimmunoprecipitation)

In accordance with prior documented procedures, Co-IP assays were performed [16, 29–31]. A detailed description of the antibodies used can be found in Table S4.

### Proximity ligation assay (PLA)

The Duolink® In Situ Red Starter Kit Mouse/Rabbit (Sigma Aldrich) was employed for PLA. The fixation of cells was conducted on glass coverslips utilizing a 4% paraformaldehyde solution. After permeabilization with 0.1% Triton X-100, the cells were exposed to a blocking step for a duration of 1 h employing a blocking solution. Subsequently, the antibodies targeting E2F6 and NBR1 (manufactured by Abcam, located in Cambridge, USA) were subjected to overnight incubation at 4 °C. Subsequently, primary antibodies were supplemented with PLA probes to facilitate binding to their respective positive or negative strands. Incubation with ligase and polymerase was performed sequentially at room temperature. Subsequently, the slides were affixed onto Duolink® In Situ Mounting Medium, which contained DAPI. Our previous article [16, 23] discussed the protocol in more detail.

### Biotin-RNA pull-down assays

In summary, the amplification of full-length LIN01592 sequences was achieved through the utilization of PCR and reversed transcription. The proteins were extracted by cell lysis employing a lysis buffer. A biotin-labeled LIN01592 probe was then captured on streptavidin agarose beads. The quantification of pulldown complexes was accomplished through either mass spectrometry or WB. A more detailed description of the protocol can be found in the previous article [23, 24].

### RIP assays

Magna RNA-binding protein immunoprecipitation kit (Millipore) was employed to perform the RIP assays. The cell was lysed after being gathered employing RIPA buffer. Subsequently, the cell lysate was subjected to incubation with RIP buffer that comprised magnetic beads that were conjugated to either human anti-E2F6 or IgG antibodies. Subsequently, the co-precipitated RNA was quantified utilizing qRT-PCR. In order to verify the association between the identified RNA signal and E2F6, measurements were taken for both total RNA and IgG controls. An additional explanation of the protocol can be found in the preceding publication [23, 24].

### Xenografts assay

The present investigation involved the acquisition of BALB/c-nude mice (4–5 weeks old, 18–20 g) from Vitalriver (Beijing, China). Different lentiviral particles were employed to transduce Eca-109 and primary EC cells (PECC). Following the selection of puromycin for a duration of 72 h, a quantity of 1 × 10^7^ cells per mouse received injections subcutaneously into the dorsal region of mice. The methodology connected to the xenograft assay has previously been clarified [16]. The measurement of xenografts' volume and mass was conducted at the investigation endpoint.

### Statistical analysis

The statistical analyses were conducted employing SPSS23 software and version 8.0 GraphPad Prism. Additional information can be accessed through the supplementary materials.

## Results

### M2-TAMs-derived exosome enhance EC immune escape

**Fig. 1:**
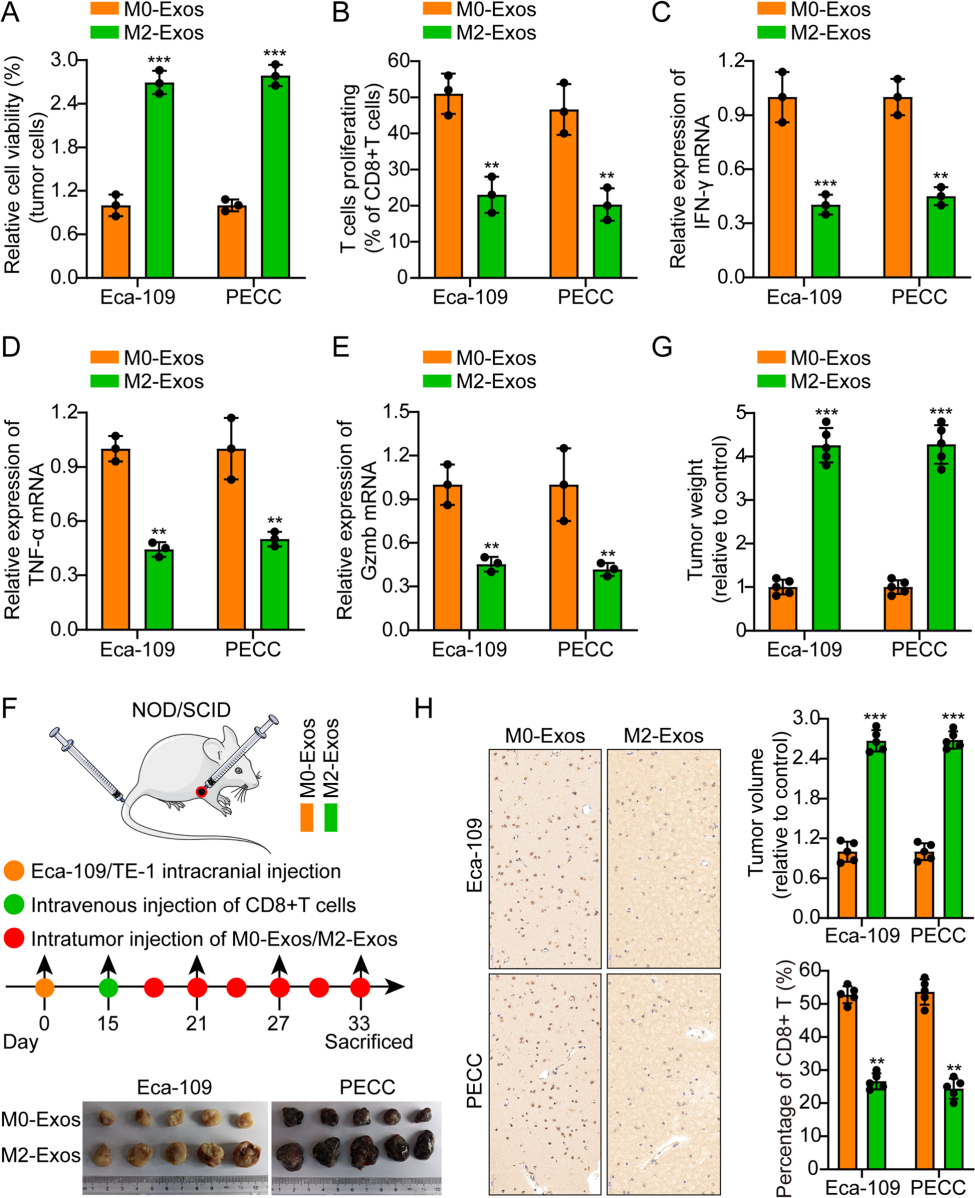
M2-TAMs-derived exosomes promote esophageal cancer immune escape. **A**. M2-Exos significantly inhibited T cell-mediated tumor cell killing compared to M0-Eoxs. **B-E**. Flow cytometry and real-time quantitative PCR results indicated that CD8^+^ T cells cocultured with M2-Exos-esophageal cancer cells showed lower proliferation and expression of IFN-γ, TNF-α, and Gzmb. **F**. Schematic diagram of animal experiments. **G-H**. Typical animal in vivo imaging pictures, typical column charts, and CD8 + IHC pictures in different groups. Scale bar, 50 µm. The means ± SDs are provided (*n* = 3). **P < 0.01 and ***P < 0.001 according to two-tailed Student t-tests or one-way ANOVA followed by Dunnett tests for multiple comparisons

Due to the macrophage characteristics of primed THP1 cells, they have been extensively employed in many studies as a model of macrophage [32–34]. Using THP1 cells polarized into M2-TAMs, we mimicked TAMs [35]. M2-TAM biomarkers CCL2, IL1RN, TGFB1, IL-10, and CD163 were found to be elevated, and M1 markers TNFA was found to be reduced through qRT-PCR (Fig. S1A). Compared to M0-Exoxs, M2-Exos significantly suppressed tumor cell killing mediated by T cells (Fig. 1A). The present study involved conducting an in vitro coculture of CD8^+^ T cells with M2-Exos-EC cell. Consequently, the growth of CD8^+^ T cells and the expression of IFN-γ, TNF-α, and Gzmb were determined by employing flow cytometry and PCR. The outcomes exhibited that CD8^+^ T cell exhibited lower TNF-α, IFN-γ, and Gzmb expression levels and growth (Figs. 1B-E). Furthermore, ELISA outcomes exhibited that CD8^+^ T-cells supernatants cocultured with M2-Exos-EC cell had lower level of IFN-γ, TNF-α, and Gzmb secretion (Fig. S1B). A subcutaneous xenograft model of nude mice was built in the following manner: Subcutaneous injection of Eca-109/PECC cells was performed in the dorsal region of mice. The mice were separated into two groups and subsequently administered M0/M2-Exos at three-day intervals following a 15-day period. Activated CD8^+^ T cells were introduced via the caudal vein, originating from the peripheral blood of healthy human donors. The findings indicated that the group of mice that received M2-Exos injections exhibited a statistically significant increase in tumor volume compared to the group that received M0-Exo injections. The IHC findings of CD8^+^ in cancer samples from transplanted animals demonstrated that the M2-Exos groups exhibited a significant decrease in this particular protein expression (Figs. 1F-G). Consistent with expectations, IF of Ki-67 in animal transplanted tumor samples revealed that mice in the M2-Exos group exhibited elevated Ki-67 expression (Fig. S1C). Furthermore, analysis of macrophage biomarkers via IF on transplanted tumor samples in each group revealed that the M2-Exos groups had an increased percentage of F4/80^+^ CD206^+^ in contrast to the control M0-Exos groups (Fig. S1D). The findings of our trial indicate that exosome obtained from M2-TAM that were isolated obviously suppressed the anti-tumor immunity mediated by CD8^+^ CTL cells in EC cells.

### LINC01592 secreted by M2-TAM is transferred to EC cell

**Fig. 2:**
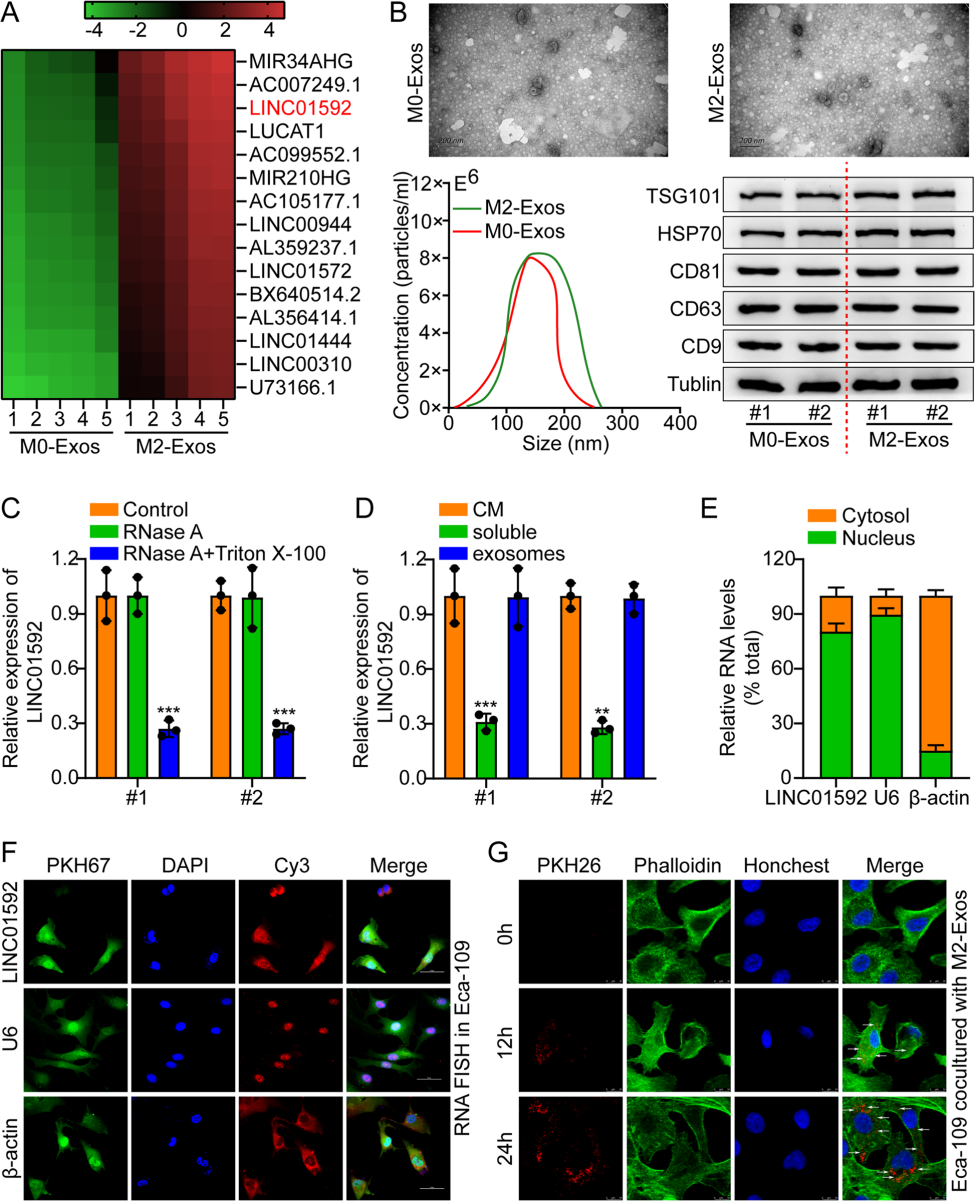
M2-TAMs-secreted LINC01592 is transferred to esophageal cancer cells. **A**. The heatmap reflected one of the upregulated lncRNAs in the RNA sequence analysis of two pairs of M0-Exos and M2-Exos. **B**. TEM, NTA, and WB were adopted to measure the morphology, size, and surface markers of exosomes. Scale bar, 200 nm. **C**. Measuring the levels of LINC01592 in CM after M2-TAM treated with RNase A alone or RNase A and Triton X-100 simultaneously. **D**. Measuring the levels of LINC01592 in CM, soluble, and exosomes. **E–F**. RNA FISH in Eca-109 and a typical column chart. Scale bar, 50 µm. **G**. PKH26-labeled M2 exosomes were added to Phalloidin-labeled Eca-109 cells. Scale bar, 25 µm. The means ± SDs are provided (*n* = 3). **P < 0.01 and ***P < 0.001 according to two-tailed Student t-tests or one-way ANOVA followed by Dunnett tests for multiple comparisons

To detect exosome-related lncRNAs in TAMs, lncRNA arrays of M0-Exos and M2-Exos were conducted. LINC01592 was identified as a prominent non-coding RNA through hierarchical clustering analysis (Fig. 2A). The morphological characteristics, dimensions, and surface biomarkers of exosomes were assessed through the utilization of NTA (Nanoparticle Tracking Analysis), TEM (Transmission Electron Microscopy), and WB (Western Blotting) techniques (Fig. 2B). Additionally, we used calnexin (exosome negative marker) to identify the exosome preparations purity (Fig. S2A). Subsequently, we examined extracellular LINC01592. M2-TAMs supplemented with RNase A alone showed no change in LINC01592 levels in a conditioned medium (CM). However, after Triton X-100 and RNase A treatment, LINC01592 levels in CM decreased (Fig. 2C). In addition, LINC01592 levels in exosomes were identical to those in the CM (Fig. 2D). On the basis of the results of this investigation, it was determined that exosomes serve as the main transporters of extracellular LINC01592.

The content of LINC01592 was detected in M0/M2-TAMs employing qRT-PCR. An elevated LINC01592 expression level was observed in the M2-TAMs in contrast to the primary M0-TAMs (Fig. S2B). LINC01592 expression was detected to be significantly higher in the four EC cell lines in contrast to Het-1A (Fig. S2C). M2-Exos were upregulated or downregulated when LINC01592 was overexpressed or knocked down in M2-TAMs, respectively (Fig. S2D). In addition, FISH and subcellular fractionation indicated that LINC01592 was situated in the nucleus and cytoplasm and mostly in the EC cells' nucleus (Figs. 2E-F). Afterward, phalloidin-labeled Eca-109 cells were subjected to incubation with PKH26-labeled exosomes. The incubated Eca-109 cells were discovered to exhibit a colocalization of phalloidin lipid dye and PKH26 fluorescence, thereby indicating the efficient absorption of exosomes (Fig. 2G). The findings indicate that LINC01592 is present within exosomes secreted by M2 cells and has the potential to be transmitted to EC cells. The expression level of LINC01592 was assessed in Eca-109 and PECC cell lines following incubation with exosome obtained from M2/Vector and M2/LINC01592-OE (M2/1592-OE). Incubation with M2/1592-OE exosomes clearly increased LINC01592 in Eca-109 and PECC cells (Fig. S2E). Exosomes from M2/1592-OE failed to increase LINC01592 in Eca-109 and PECC cells after Annexin V treatment (Fig. S2F). LINC01592 secreted by M2 was transported to EC cells by exosomes.

### M2-TAM-derived exosome LINC01592 induces tumor immune escape

**Fig. 3:**
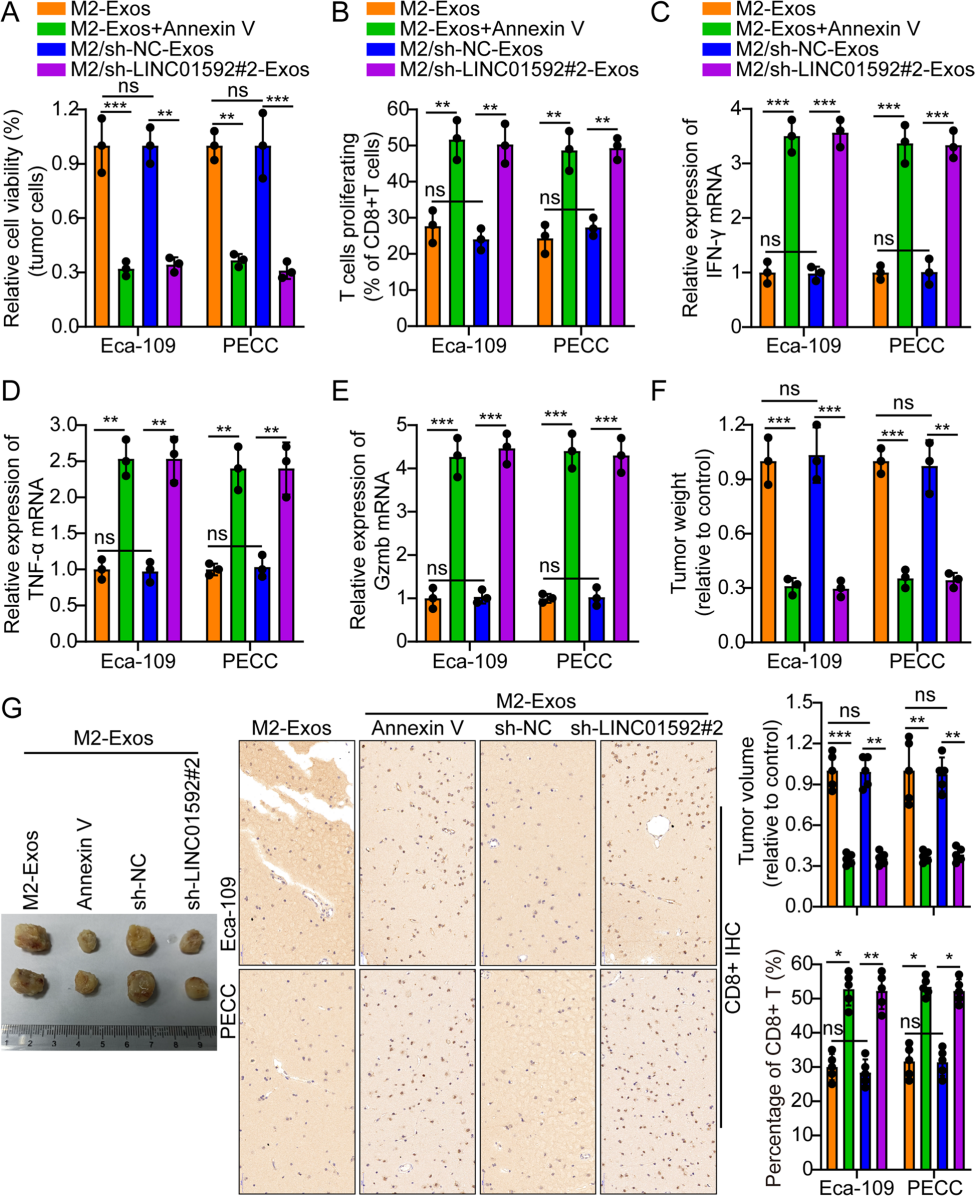
M2-TAM-derived exosomal LINC01592 induces tumor immune escape. **A**. M2-Exos significantly inhibited T cell-mediated tumor cell killing compared to M0-Eoxs. However, after adding an exosome inhibitor Annexin V or knocking down LINC01592, the above phenomenon was restored **B-E**. Flow cytometry and real-time quantitative PCR results indicated that CD8^+^ T cells cocultured with M2-Exos-esophageal cancer cells showed lower proliferation and expression of IFN-γ, TNF-α, and Gzmb. However, after adding an exosome inhibitor Annexin V or knocking down LINC01592, the above phenomenon was restored. **F-G**. Typical animal in vivo imaging pictures, typical column charts, and CD8^+^ IHC pictures in different groups. Scale bar, 50 µm. The means ± SDs are provided (*n* = 3). **P < 0.01 and ***P < 0.001 according to two-tailed Student t-tests or one-way ANOVA followed by Dunnett tests for multiple comparisons. ns, no significant difference

LINC01592 expression level in EC and its association with patient outcome were first determined using the TCGA database. According to the results, LINC01592 was greatly expressed in EC, and its level of expression was reversely related to the prognosis (Fig. S3A). A lentiviral vector comprising various siRNAs and two shRNAs was used to knock down the LINC01592 gene. In our investigation, the shRNA sh-1592#2 revealed the strongest LINC01592 suppression (Fig. S3B). The coculture outcomes indicated that T-cell-mediated tumor cells killing was significantly inhibited by M2-Exos in comparison to M0-Eoxs. The observed phenomenon was reversed subsequently to the introduction of Annexin V or the downregulation of LINC01592 (Fig. 3A). The investigation involved coculturing of CD8^+^ T cells with M2-Exos-EC cell in vitro. The subsequent measurement of CD8^+^ T cell growth and expression of IFN-γ, TNF-α, and Gzmb was conducted employing flow cytometry and PCR. The findings indicated that CD8^+^ T cell demonstrated reduced growth and decreased IFN-γ, TNF-α, and Gzmb expression. Nevertheless, the aforementioned phenomenon was reversed subsequently to the introduction of Annexin V or the knocking down of LINC01592 (Figs. 3B-E).

Additionally, the ELISA outcomes indicated that the supernatants of CD8^+^ T-cells that were co-cultivated with M2-Exos-EC cells exhibited decreased IFN-γ, TNF-α, and Gzmb secretion levels. Nevertheless, subsequent to the introduction of Annexin V or the depletion of LINC01592, this phenomenon exhibited a reversal (Fig. S3C**)**. The nude mouse subcutaneous xenograft model was established as follows: Eca-109/PECC cells were injected into the back of mice subcutaneously. The mice were distributed into four groups after 15 days, and the tumor was injected every three days with M2-Exos + Annexin V, M2-Exos, M2/sh-1592#2-Exos, and M2/sh-NC-Exos. Through the tail vein, activated CD8^+^ T cells were injected. Our results suggest that M2/sh-1592#2-Exos or M2-Exos + Annexin V obviously decreased the tumor volume in contrast to mice subjected to administration of M2/sh-NC-Exos or M2-Exos alone. The results of IHC of CD8^+^ in animal transplanted tumor samples indicate that mice administered with M2/sh-1592#2-Exos or M2-Exos + Annexin V exhibited considerably elevated levels of the protein in comparison to mice administered with M2/sh-NC-Exos or M2-Exos alone (Figs. 3F–G). Furthermore, the IF of Ki-67 in animal transplanted cancer samples revealed that the group of mice administered with M2/sh-1592#2-Exos or M2-Exos + Annexin V exhibited reduced expression of this protein compared to the group of mice administered with M2/sh-NC-Exos or M2-Exos alone (Fig. S3D). Additionally, IF analysis was conducted on macrophage biomarkers in transplanted cancer samples from each group of animals. The findings indicate that the percentage of F4/80^+^ CD206^+^ in the M2-Exos + Annexin V or M2/sh-1592#2 Exos groups were obviously lower compared to the control groups M2/sh-NC-Exos or M2-Exos (Fig. S4A). Furthermore, it was observed that the expression of LINC01592 exhibited an inverse correlation with the infiltration of CD8^+^ T-cells in the TCGA database (Fig. S4B). The experimental findings suggest that the LINC01592 derived from M2-Exos is responsible for inducing immune evasion in tumors. In contrast, these mechanisms were not enhanced by exosomes that were subjected to Annexin V treatment or LINC01592 knockdown.

### LINC01592 accelerates the degradation of MHC-1 through the autophagy-lysosome pathway

**Fig. 4:**
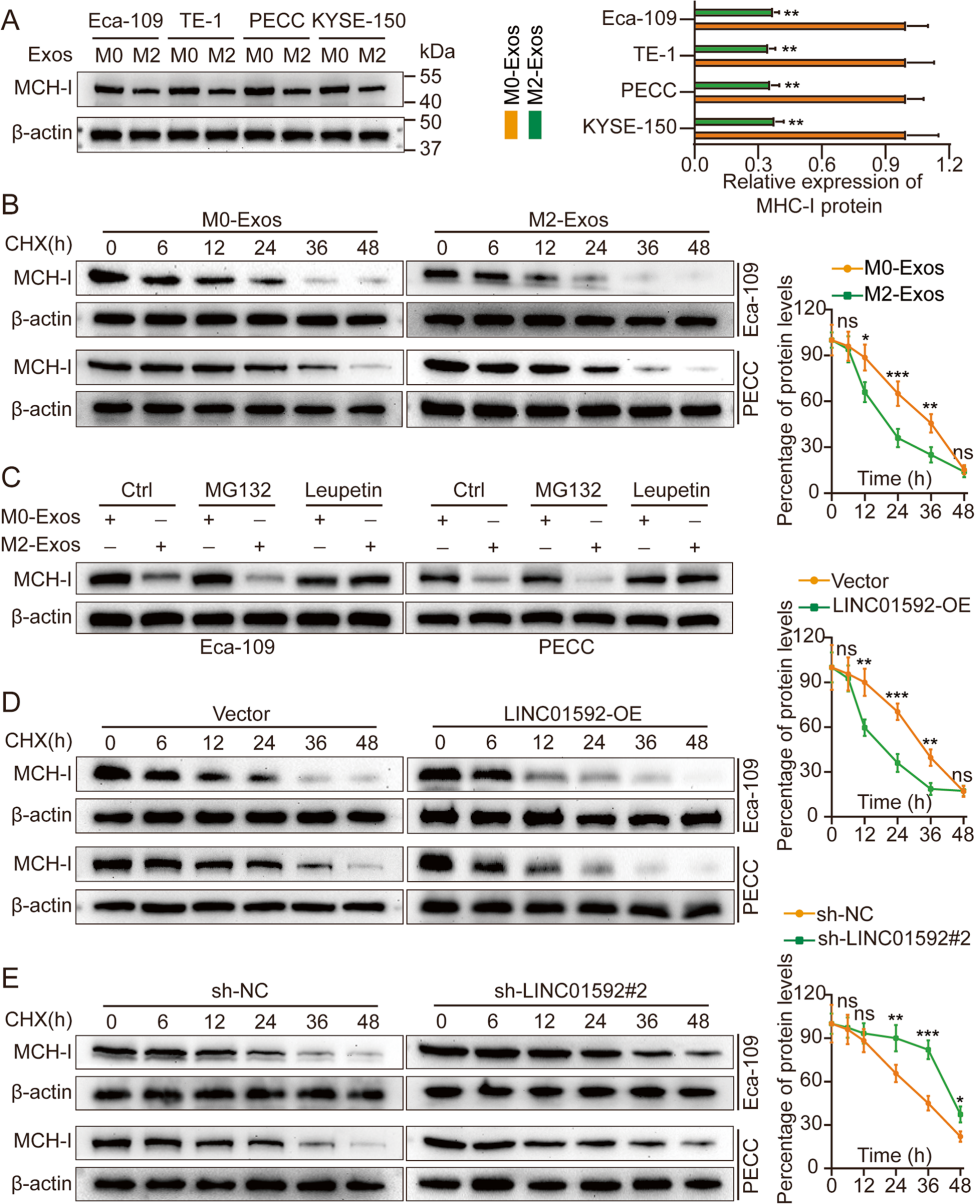
M2-Exos-derived LINC01592 mediates MHC-I expression by regulating the autophagy-lysosomal pathway. **A**. Different tumor cell lines were treated with M0-Exos and M2-Exos, and the expression level of MHC-I was detected by WB. The right side represents a typical column chart. **B**. Eca-109/PECC cells were treated with M0-Exos and M2-Exos, and the expression level of MHC-I was detected by WB using CHX assay. The right side represents a typical protein degradation curve. **C**. Eca-109/PECC cells with M0/M2-Exos simultaneously with the proteasome inhibitor MG132 and the lysosomal inhibitor Leupetin, and the expression level of MHC-I was detected by WB. **D-E**. Knockdown and overexpression of LINC01592 in Eca-109/PECC, respectively, and the expression level of MHC-I were detected by WB using CHX assay. The right side represents a typical protein degradation curve The means ± SDs are provided (*n* = 3). *P < 0.05, **P < 0.01, and ***P < 0.001 according to two-tailed Student t-tests or one-way ANOVA followed by Dunnett tests for multiple comparisons. ns, no significant difference.

Following activation, CD8^+^ T lymphocytes undergo differentiation into cytotoxic T cells (CTLs). The primary mechanism responsible for activating CTL in the immune response against tumors is the creation of the MHC-I-antigen peptide-TCR complex. Tumor cells have the ability to evade immune attack by CTLs due to alterations or deficiencies in MHC-I molecules present on their surface [36, 37]. Interestingly, in comparison to M0-Exos, M2-Exos did not significantly affect the level or stability of MHC-I mRNA in EC cells. However, they significantly reduced the levels and stability of the MHC-I protein (Figs. 4A-B and S5A-B). Therefore, it is hypothesized that exosomes originating from M2-TAM enhance the MHC-I protein in EC cell degradation as a mechanism to evade anti-tumor immunity mediated by CD8^+^ T-cells.

There are two main pathways by which intracellular proteins are degraded: the autophagy-lysosome and ubiquitin–proteasome pathways. The ubiquitin–proteasome mechanism is primarily responsible for the degradation of numerous short-lived proteins, and it employs temporal regulation to precisely adjust the steady-state level of various modulatory protein [38]. The autophagy-lysosome pathway is primarily responsible for the degradation of long-lived proteins and certain organelles located within the cytoplasm [39]. In this investigation, EC cells were subjected to treatment with M0/M2-Exos, followed by co-treatment with MG132 (a proteasome suppressor) and leupeptin (a lysosomal suppressor) at a concentration of 10 μM for 8 h. The findings indicated that only the lysosomal suppressor was able to eliminate the suppressive impact of M2-Exos on MHC-I (Fig. 4C). The half-life of MHC-I was observed to be shorter in CHX (cycloheximide chase assay) in cells that were overexpressing LINC01592. Similarly, the MHC-I exhibited an extended half-life in cells that were undergoing downregulation of LINC01592 (Figs. 4D-E). To explore the impact of MHC-I expression on lysosomes, IF experiments were carried out utilizing anti-LAMP1 and anti-MHC-I antibodies. Compared to controls, overexpression of LINC01592 attenuated MHC-I expression in lysosomes, whereas knockdown of the gene increased it (Fig. S5C). After overexpressing LINC01592, cells were treated with chloroquine (CQ, the autophagy suppressor) at a concentration of 50 μM for 10 h and leupeptin (the lysosomal suppressor) at a concentration of 10 μM for 8 h, and levels of MHC-I protein were measured by WB. LINC01592 could be rescued by CQ and leupeptin to promote the degradation of MHC-I (Fig. S6A). qRT-PCR was used to confirm LINC01592 overexpression (Fig. S6B). By modulating the autophagy-lysosome mechanism, LINC01592, originating from M2-Exos, induces tumor immune escape by mediating MHC-I expression.

### LINC01592 binds to E2F6 and accelerates its nuclear translocation of E2F6

**Fig. 5:**
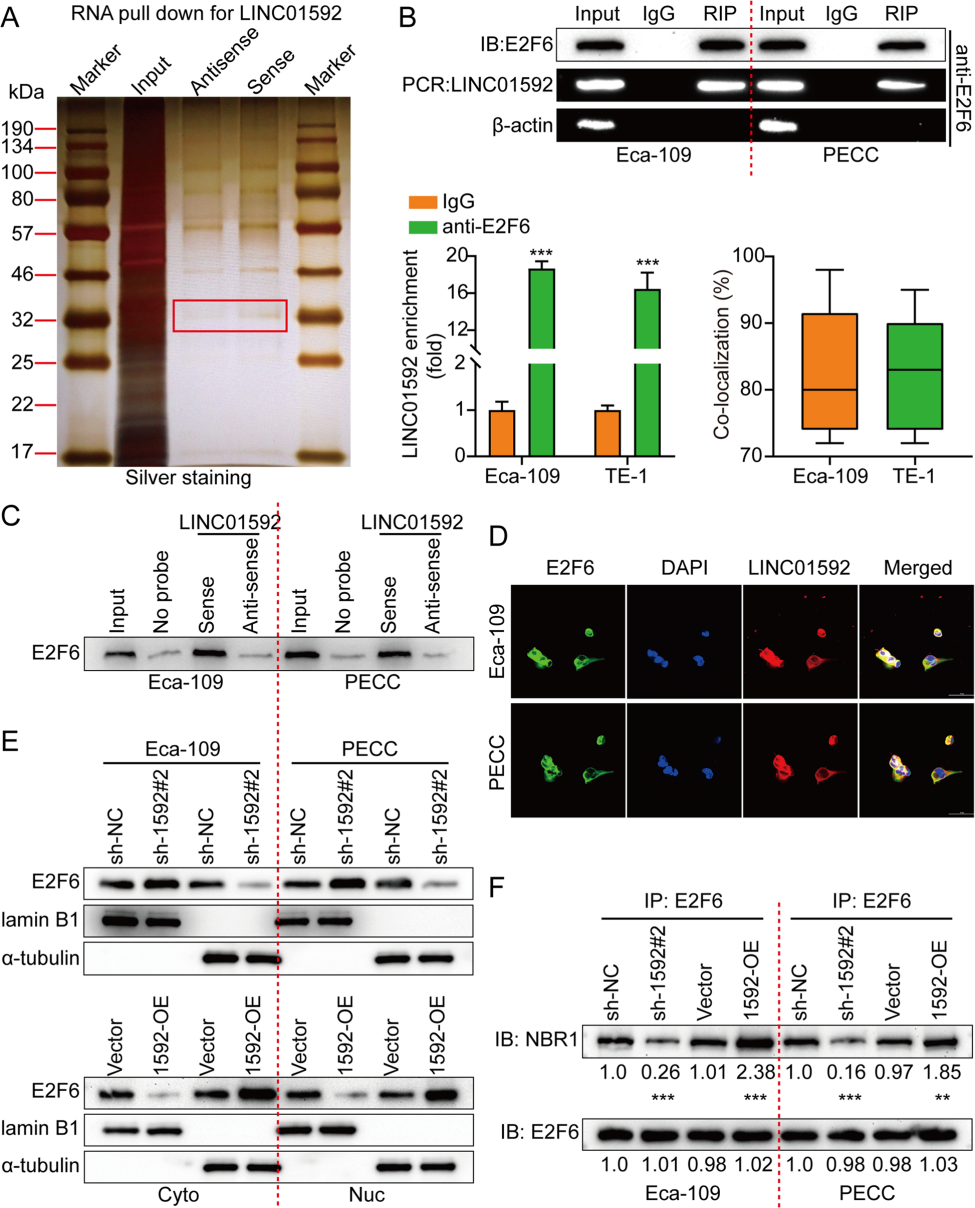
LINC01592 binds with E2F6 and accelerates the nuclear translocation of E2F6. **A**. The protein extracted from LINC01592 pulldown assays was analyzed by MS. **B**. According to RIP assays with anti-E2F6 antibodies, E2F6 interacted with LINC01592 in Eca-109/PECC. Top, agarose electrophoresis of PCR product. Bottom, qPCR result of RIP assay. **C**. The antisense sequences of LINC01592 served as negative controls for the WB detection of the protein obtained from LINC01592 pulldown assays. **D**. Representative images of E2F6 subcellular localization in Eca-109 under different treatments are shown in IF staining. Anti-E2F6 (red) and DAPI (blue) were used to stain all cells. Analysis of the average fluorescence intensity of E2F6 and DAPI signals was conducted using ImageJ. **E**. WB analysis of Eca-109 nuclear and cytosolic lysates was performed after knockdown or overexpression of LINC01592. **F**. Co-IP assays were performed on LINC01592 knockdown, LINC01592 overexpression, and control groups to determine the E2F6–NBR1 interaction. The means ± SDs are provided (*n* = 3). **P < 0.01 and ***P < 0.001 according to two-tailed Student t-tests.

The study conducted RNA pulldown and MS to detect proteins that interact with LINC01592. E2F6 was determined through RNA pulldown analysis (Fig. 5A) and MS outcomes (Fig. S6c). The RIP assay revealed a specific enrichment of LINC01592 within the complexes immunoprecipitated by E2F6 (Fig. 5B). According to the findings of the LINC01592 pulldown assays, it has been observed that E2F6 exhibits a specific binding affinity to the sense strand, while no such binding has been detected with the antisense strand (Fig. 5C).

Based on the anticipated secondary construction utilizing the RNAfold web server (http://rna.tbi.univie.ac.at//cgi-bin/RNAWebSuite/RNAfold.cgi), we constructed four truncations of LINC01592 to validate its binding to E2F6. The LINC01592 gene was found to contain E2F6-specific binding sequences within the 594–1186 nt region through the utilization of RNA pulldown assays. The protein's interaction domains were identified through the employing of RIP assays with Flag-tagged E2F6 truncations. LINC01592 was bound by the A2 domain (143–236) of E2F6 (Figs. S6D-E). Moreover, LINC01592 modulates the expression of E2F6 in both the nuclear compartment and the entire cellular context. In Eca-109/PECC negative control cells, most E2F6 was found in the cytoplasm. In LINC01592 knockdown cells, E2F6 was almost undetectable. On the other hand, LINC01592-overexpressing cells had a significant amount of E2F6 in their nuclei (Fig. 5D). Cytoplasmic lysates from Eca-109/PECC cells had the highest levels of E2F6 compared to the nuclear lysate. The study found that the knockdown of LINC01592 in Eca-109/PECC cells resulted in a significant decrease in the levels of E2F6 in both the nucleus and cytoplasm. Following ectopic expression of LINC01592, nucleus-associated E2F6 levels increased (Fig. 5E).

By binding to promoters, E2F6 can inhibit or activate gene transcription. Shao G et al. reported that E2F6 could promote breast cancer progression by upregulating PNO1 by binding to the PNO1 promoter [40]. The present study suggested that LINC01592 could promote the binding between E2F6 and the promoter region of NBR1. The gene is located in a region of chromosome 17q21.1 that is very close to the BRCA1 tumor suppressor gene. The protein encoded by this gene was originally identified as an ovarian tumor antigen monitored in ovarian cancer. The encoded protein contains a B-box/coiled-coil motif that is present in many genes with transforming potential. It acts as a specific autophagy receptor for selective autophagic degradation by peroxisomes by forming intracellular envelopes with ubiquitinated autophagy substrates. A recent study shows that NBR1 binds to MHC-I through its ubiquitin-binding domain and mediates the degradation of MHC-I molecules in autophagy lysosomes, thereby promoting immune evasion of pancreatic cancer. First, we tested whether LINC01592 could mediate the mutual binding between E2F6 and NBR1 using Co-IP. This binding was enhanced by overexpression of LINC01592 and weakened by knockdown (Fig. 5F). Additionally, PLA was used to visualize natural protein complexes. The downregulation of LINC01592 resulted in a reduction in protein complexes that exhibit PLA positivity, namely E2F6 and NBR1. Furthermore, up-regulation of LINC01592 caused the construction of highly dense clusters of E2F6/NBR1, consistent with the findings of Co-IP analysis (Fig. S7A). Additionally, knockdown and overexpression of LINC01592 did not affect the levels of E2F6 mRNA or protein (Figs. S7B-C). The findings indicate that LINC01592 has the ability to bind to E2F6 while promoting its translocation to the nucleus. Mechanistically, it is still unclear how LINC01592 induced E2F6 localization into the nucleus. It is well known that LINC01592 influences the ubiquitination or degradation of E2F6 protein, which is a crucial step for its nuclear translocation. Next, we knocked down and overexpressed LINC01592 respectively, and treated it with the proteasome inhibitor MG132. Finally, we used WB to detect the expression level of E2F6 in the cytoplasm. The results showed that knockdown of LINC01592 down-regulated the expression level of E2F6 in the cytoplasm, while overexpression of LINC01592 up-regulated the expression level of E2F6 in the cytoplasm. The recovery experiment showed that MG132 can rescue the down-regulated expression level of E2F6 in the cytoplasm of LINC01592 (Figs. S13A). As expected, in the cycloheximide chase assays (CHX), E2F6 had a shorter half-life in cells overexpressing LINC01232; similarly, in cells downregulating LINC01592, E2F6 had a longer half-life (Figs. S13B). Furthermore, LINC01592 silencing increased E2F6 ubiquitination in the Eca-109 and PECC cells (Figs. S13C).

### LINC01592 promotes E2F6-mediated transcription of NBR1

**Fig. 6:**
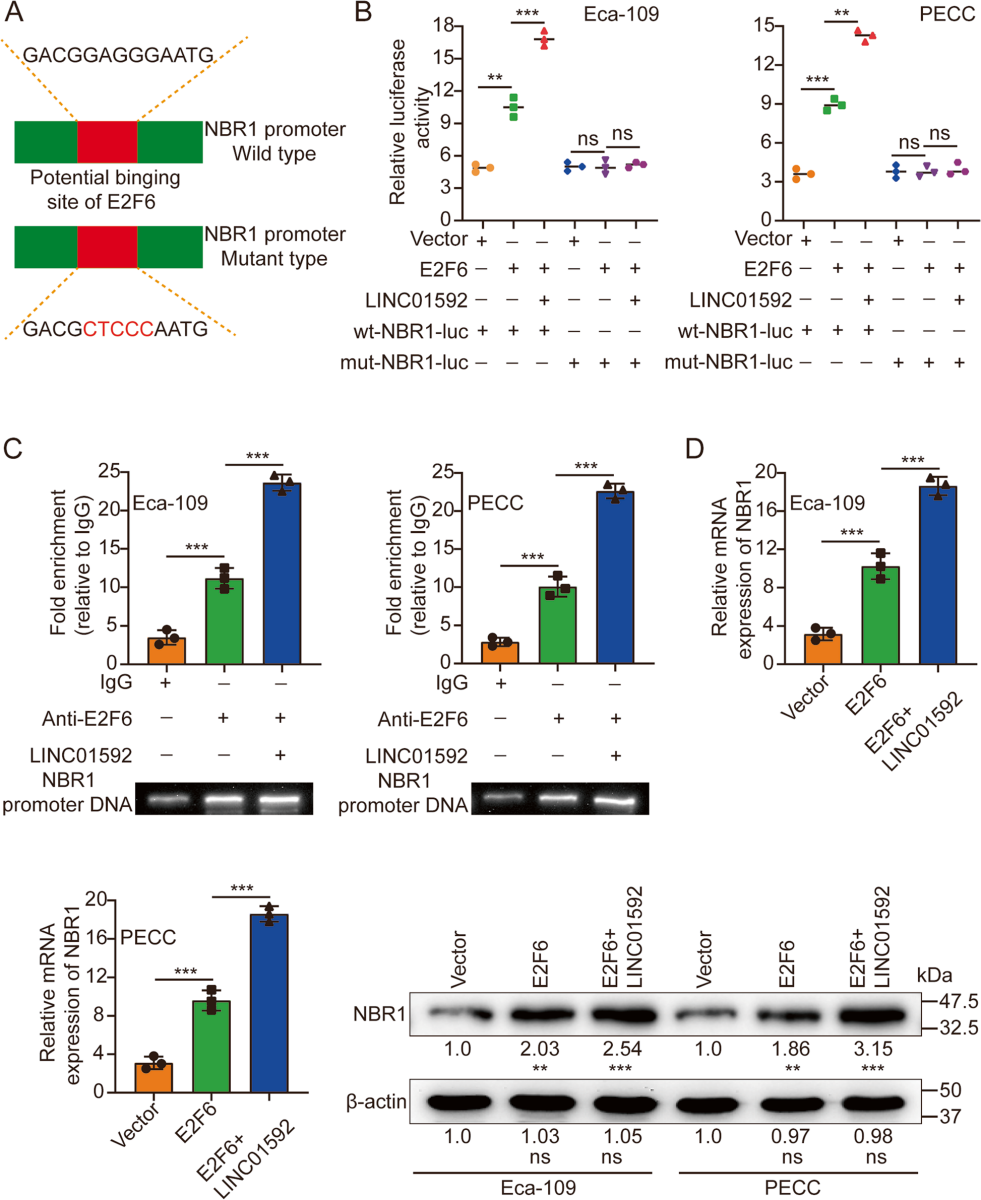
LINC01592 promotes E2F6-mediated transcription of NBR1. **A**. We constructed the wild-type or mutant-type luciferase vectors based on the potential binding site of E2F6 to the NBR1 promoter. **B**. Luciferase activity was assayed in Eca-109/PECC cells transfected with luciferase vectors (wild type or mutant type) and, meantime, co-transfected with expression plasmids (empty vectors, E2F6 expression plasmids, or LINC01592 expression plasmids). **C**. ChIP, experiments of E2F6 (IgG as an internal control), were performed, and the co-precipitated DNA was subjected to PCR amplification with primers specific to the NBR1 promoter region. **D**. The level of NBR1 under ectopic expression of E2F6 or LINC01592 was detected by qRT-PCR and western blotting. The means ± SDs are provided (*n* = 3). **P < 0.01 and ***P < 0.001 according to two-tailed Student t-tests or one-way ANOVA followed by Dunnett tests for multiple comparisons. ns, no significant difference

Transcription factors exhibit binding affinity to particular DNA sequences in order to modulate the gene's expression. Through the utilization of the JASPAR database [41], it was determined that the binding of E2F6 to the promoter of NBR1 was observed. To establish that NBR1 is a transcriptional target of E2F6 and that LINC01592 could enhance its modulation, luciferase vectors containing the WT or mutated NBR1 promoters were constructed and subsequently transfected into Eca-109/PECC cells (Fig. 6A). The luciferase assay demonstrated that the upregulation of E2F6 resulted in the stimulation of WT NBR1 promoter activity, as evidenced by an elevation in luciferase activity. However, overexpression did not affect the mut NBR1 promoter activity. Additionally, LINC01592 enhanced E2F6-induced luciferase activity (Fig. 6B). Furthermore, it was demonstrated through ChIP assays that the binding of E2F6 to the NBR1 promoter was observed, and this interaction was promoted by LINC01592 (Fig. 6C). The upregulation of E2F6 resulted in a significant rise in both NBR1 mRNA and protein levels, which was subsequently promoted by LINC01592 (Fig. 6D). The RNA pulldown and RIP assays did not provide evidence of direct interaction between LINC01592 and NBR1 (Figs. S7D-E). As hypothesized, our findings indicated that the mutation of the binding site of E2F6 on ΔLINC01592 did not result in an acceleration of the MHC-I proteins degradation. (Fig. S7F); strengthen the binding of E2F6 to NBR1 (Fig. S7G) or advance the nuclear translocation of E2F6 (Fig. S8A). Additionally, we discovered that ΔLINC01592 did not promote E2F6-induced luciferase activity (Fig. S8B), strengthen E2F6 binding to NBR1's promoter (Fig. S8C), or reinforce E2F6 upregulation of NBR1 mRNAs and proteins (Fig. S8D). The outcomes of our investigation indicated that E2F6 has the capability to bind directly to the promoter region of NBR1, consequently facilitating its transcriptional activation. Additionally, LINC01592 functions to strengthen this mechanism.

### LINC01592 promotes EC immune escape by regulating NBR1

**Fig. 7:**
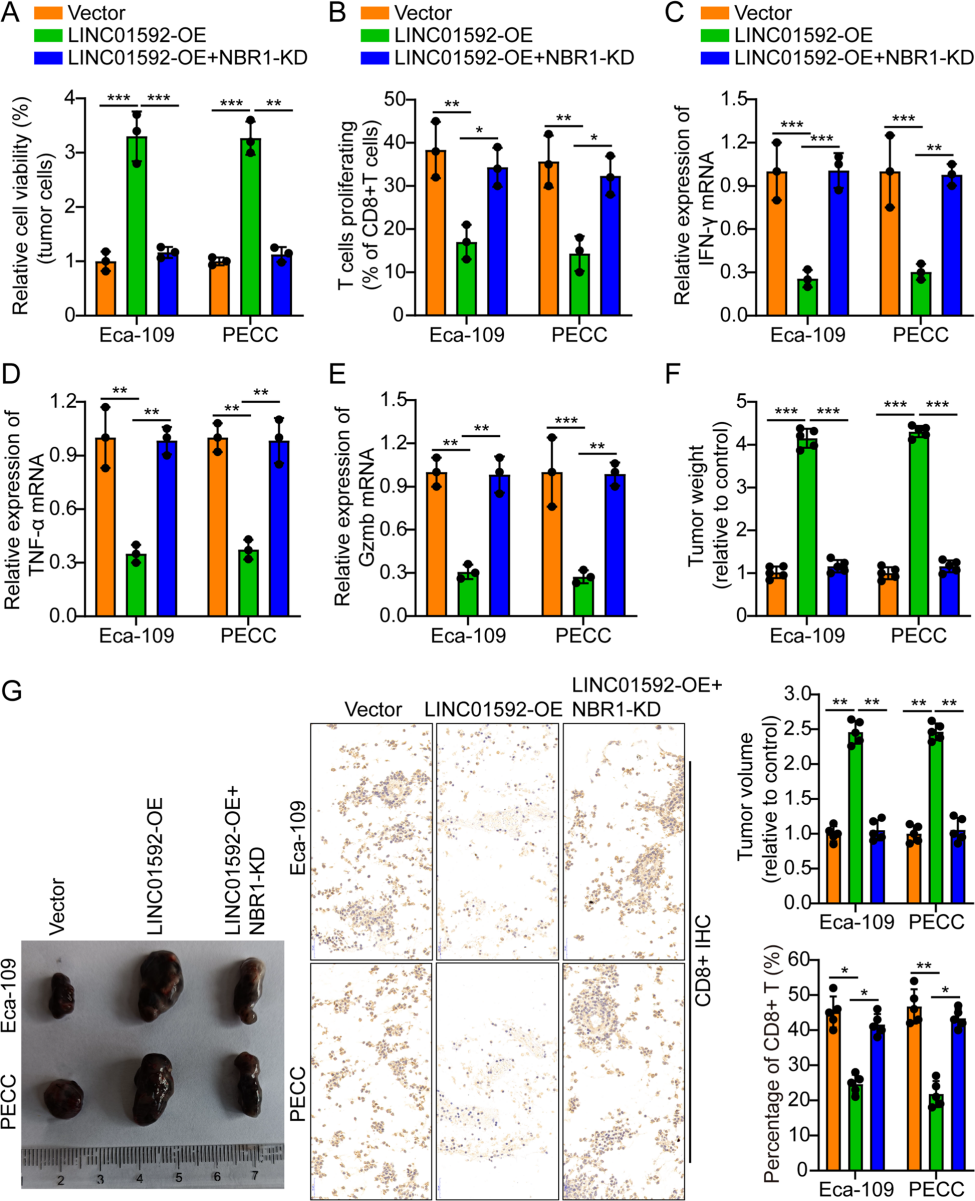
LINC01592 promotes esophageal cancer immune escape by regulating NBR1. **A**. LINC01592-OE/Eca-109/PECC cells significantly inhibited T cell-mediated tumor cell killing compared to Vector; however, knocking down NBR1 while overexpressing LINC01592 could rescue the above phenomenon. **B-E**. Flow cytometry and real-time quantitative PCR results indicated that CD8^+^ T cells cocultured with LINC01592-OE-esophageal cancer cells showed lower proliferation and expression of IFN-γ, TNF-α, and Gzmb; however, knocking down NBR1 while overexpressing LINC01592 could rescue the above phenomenon. **F-G**. Typical animal in vivo imaging pictures, typical column charts, and CD8^+^ IHC pictures in different groups. Scale bar, 50 µm. The means ± SDs are provided (*n* = 3). *P < 0.05, **P < 0.01, and ***P < 0.001 according to two-tailed Student t-tests or one-way ANOVA followed by Dunnett tests for multiple comparisons. ns, no significant difference

To detect the role of NBR1 modulation in the enhancement of EC immune evasion by LINC01592, we knocked down NBR1 in the EC cells Eca-109/PECC while overexpressing LINC01592. We then utilized WB to validate NBR1 knockdown efficacy (Fig. S8E). The findings of the cell coculture experiment indicated that LINC01592 in Eca-109/PECC cells resulted in significant suppression of T-cell-mediated tumor cell killing. Nevertheless, the observed phenomenon was reversed by downregulating NBR1 and upregulating LINC01592 (Fig. 7A). The study involved conducting an in vitro coculture of CD8^+^ T cells with LINC01592-OE EC cells, followed by the use of flow cytometry and PCR to measure the growth of CD8^+^ T cells and the expression of IFN-γ, TNF-α, and Gzmb. The outcomes indicated that CD8^+^ T cells exhibited lower growth and IFN-γ, TNF-α, and Gzmb expression levels. Nevertheless, the observed phenomenon was reversed by downregulating NBR1 and upregulating LINC01592 (Figs. 7B-E).

Furthermore, the ELISA findings demonstrated that CD8^+^ T cells supernatants that were cocultured with LINC01592-OE EC cell exhibited lower level of IFN-γ, TNF-α, and Gzmb secretion. Nevertheless, the phenomenon was reversed by downregulating NBR1 and upregulating LINC01592 (Fig. S9A). The model of nude mouse subcutaneous xenograft was built as following: Vector/LINC01592-OE/LINC01592-OE + NBR1-KD Eca-109/PECC cells were subcutaneously administered to the dorsal region of mice. The activated CD8^+^ T cells were injected through the tail vein after 15 days. A significant increase in tumor volume was identified in mice injected with LINC01592-OE. According to IHC results of CD8^+^ in animal transplanted cancer samples, mice in the LINC01592-OE groups expressed obviously less CD8^+^; however, the phenomenon could be reversed by knocking down NBR1 while overexpressing LINC01592 (Figs. 7F-G). IF outcomes related to Ki-67 in the transplanted cancer samples of animals revealed that the mice belonging to the LINC01592-OE group exhibited elevated levels of Ki-67 expression in contrast to the mice belonging to the vector group; however, knocking down NBR1 while overexpressing LINC01592 could weaken this phenomenon (Fig. S9B-S10A). These outcomes further illustrated that LINC01592 enhanced EC immune escape by modulating NBR1.

### Correlation of the LINC01592/E2F6/NBR1/MHC-I axis with clinical development

**Fig. 8:**
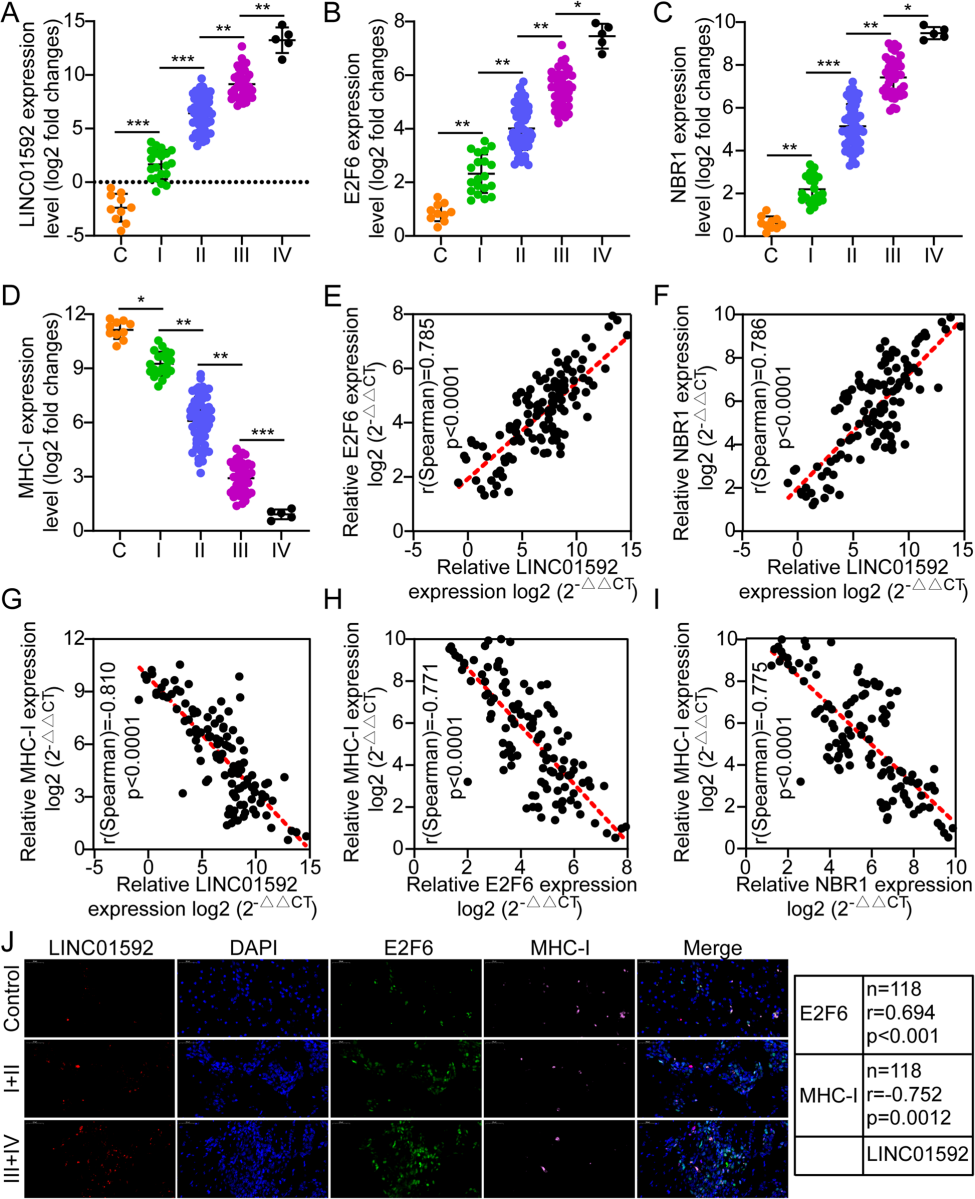
Correlation of LINC01592/E2F6/NBR1/MHC-I axis with clinical progression. **A-D**. Expression levels of LINC01592/E2F6/NBR1/MHC-I in NEE and different grades of esophageal cancer by qRT-PCR. **E**. Spearman correlation analysis between LINC01592 levels and E2F6 levels in tumor tissues from esophageal cancer patients. Pearson's correlation coefficient (r) and *P*-value, as the picture showed, *n* = 118. The *P*-value was from Spearman's test. **F**. Spearman correlation analysis between LINC01592 levels and NBR1 levels in tumor tissues from esophageal cancer patients. Pearson's correlation coefficient (r) and *P*-value, as the picture showed, *n* = 118. The *P*-value was from Spearman's test. **G**. Spearman correlation analysis between LINC01592 levels and MHC-I levels in tumor tissues from esophageal cancer patients. Pearson's correlation coefficient (r) and P-value, as the picture showed, *n* = 118. The *P*-value was from Spearman's test. **H**. Spearman correlation analysis between E2F6 levels and MHC-I levels in tumor tissues from esophageal cancer patients. Pearson's correlation coefficient (r) and *P*-value, as the picture showed, *n* = 118. The *P*-value was from Spearman's test. **I**. Spearman correlation analysis between NBR1 levels and MHC-I levels in tumor tissues from esophageal cancer patients. Pearson's correlation coefficient (r) and *P*-value, as the picture showed, *n* = 118. The P-value was from Spearman's test. **J**. Spearman correlation analysis between Linc01060 expression levels and E2F6, NBR1, and MHC-I expression levels in esophageal cancer tissues. Pearson's correlation coefficient (r) and *P*-value, as the picture showed; the P-value was from Spearman's test. The scale bar represents 50 μm. The means ± SDs are provided (*n* = 3). *P < 0.05, **P < 0.01, and ***P < 0.001 according to two-tailed Student t-tests or one-way ANOVA followed by Dunnett tests for multiple comparisons. ns, no significant difference

The expression level of LINC01592/E2F6/NBR1/MHC-I among EC and normal esophageal epithelium (NEE) tissues of different grades were compared. A higher level of LINC01592/E2F6/NBR1 expression was detected in cancer tissues, especially in III + IV. On the other hand, MHC-I expression exhibited a contrary pattern (Figs. 8A-D). The TCGA database analysis also revealed that expression of NBR1 and E2F6 is elevated in cancer tissues, and their expression was inversely related to patient outcome (Figs. S9C). In addition, we knocked down and overexpressed LINC01592 in various esophageal cancer cell lines, and used WB to detect the expression level of MHC-I. The results also showed that knockdown of LINC01592 up-regulated the expression level of MHC-I, while overexpression of LINC01592 down-regulated the expression level of MHC-I (Figs. S10B). Furthermore, we discovered that E2F6 expression was inversely related to immune cell CD8^+^ T cell infiltration (Fig. S11A). Moreover, there was a positive association between the expression levels of the signaling axis of LINC01592/E2F6/NBR1. MHC-I expression, however, was inversely proportional to LINC01592/E2F6/NBR1 (Figs. 8E-I). Furthermore, IF, FISH, and IHC outcomes showed that LINC01592 levels were positively associated with E2F6/NBR1 signaling. MHC-I expression, however, was inversely proportional to LINC01592/E2F6/NBR1 (Figs. 8J and S11B).

ROC analysis was conducted to evaluate the pathological and prognostic significance of LINC01592. The analysis involved a comparison of a LINC01592-based model, a TNM-based model, and a combination model for predicting medical outcomes. The combined model exhibited better performance (AUC = 0.769) compared to the TNM-based model alone (AUC = 0.682), as determined by the area under the curve. Utilization of LINC01592 in conjunction with the TNM stage demonstrated superior prognostic capabilities in predicting clinical outcomes compared to the use of the TNM stage in isolation (Fig. S11C). Our study also studied the connection between LINC01592 mRNA level and clinicopathological features of 118 EC specimens. Table S1 displays the relationship between the mRNA expression level of LINC01592 and the clinical, pathological, and molecular features of the tumor. The study findings indicated a significant association between the mRNA expression level of LINC01592 and various clinical parameters, including TNM stage (*P* = 0.017), T stage (*P* = 0.0003), lymph node metastasis (*P* = 0.024), distant metastasis (*P* = 0.03), mortality (*P* = 0.005), and tumor size (*P* = 0.0006) (Fig. S11D). Additionally, the outcomes of both univariate and multivariate Cox regression analyses demonstrated that elevated levels of LINC01592 mRNA expression were a significant independent predictor of unfavorable prognosis among individuals with EC (Table S2). Table S2 presents the correlation between the expression of LINC01592 mRNA and TNM stage, as well as tumor size. Collectively, these findings showed a positive connection between LINC01592 expression and clinical EC malignant grade while also revealing a negative relationship between LINC01592 expression and patient prognosis.

## Discussion

Tumor incidence and progression is a complicated process, which is also modulated by the induction of genetic mutations of cancer cells themselves and the surrounding microenvironment. Previous investigations have established that TME may be involved in a broad way in the induction of tumorigenesis and the promotion of tumor development [42]. In TME, the infiltration and activation of immune cells are biomarkers of most cancer development. Macrophages connected to tumors, as an important subset of several tumor-infiltrating immune cells, have a vital function in the interaction between the malignant cells and immunity. Macrophages, related to tumors, are a type of immune cell abundant in solid tumors [5, 6]. The infiltration of tumor-related macrophages and higher levels of related gene expression significantly impact the prognosis and therapeutic efficacy of malignancies in the majority of human cases [5]. Research findings indicate that upon phagocytosis of apoptotic tumor cells, macrophages incorporate tumor cell DNA into their nucleus, and these macrophages will be affected by tumor cell DNA and transform into similar tumorigenic cells. Cells originated from tumor stem cells, but on such cell surfaces, they still contain macrophage markers. Researchers call this type of macrophage, which is affected by the DNA of tumor cells and acquires tumor stem cell-like properties, tumor macrophage [43]. Several previous studies show that the number and function of tumor-associated macrophages directly affect the efficacy of anti-tumor therapy [44]. Currently, TAM-targeted strategies for treating tumors are divided mainly into two categories [44]: (1) Inhibition of tumor-promoting TAMs, including inhibition of their production, recruitment, and promotion of depletion. Blocking signaling mechanisms such as CCL2/CCR2 and CXCL12/CXCR4 are known to suppress monocyte/macrophage recruitment, thus inhibiting their function. An additional illustration involves the depletion of TAMs through the use of CSF-1/CSF-1R or the induction of TAM apoptosis through pharmacological agents such as bisphosphonates and trabectedin [45, 46]. (2) The activation of anti-tumor TAMs is related to the process of converting macrophages with pro-tumor features into macrophages with anti-tumor properties. For example, tumor-promoting TAMs can be reprogrammed into tumor-suppressor TAMs by PI3K-γ suppressors, CD40 agonists, anti-CD47 antibodies, and class-IIa HDAC suppressors [47, 48].

Exosomes are vesicles that originate from endosomes and have a significant function in intercellular communication. These vesicles are secreted in different biological fluids such as ascites, serum, urine, saliva, and cerebrospinal fluid [49]. Exosomes typically range in size from 30 to 150 nm in diameter and possess a vesicular morphology characterized by a bilayered membrane of biological origin. The membrane structure is mainly composed of a phospholipid bilayer (DNA, mRNA, miRNA, lncRNA, and cirRNA) [50–52]. Cells can fuse with adjacent or distant cells by secreting exosomes, mediate the transport of biologically active substances and the transmission of biological signals, and then regulate the physiological functions of cells, preserve the internal environment homeostasis and the information between cells, communicate with substances, and different contents in exosomes determine that exosomes play different biological functions [53–55]. In normal cells, the function of exosomes is to remove unfavorable biomolecules, but in cancer cells, this may promote the incidence and progression of malignancy and even affect cancer treatment. Current research shows that exosomes are strictly connected to the tumors' incidence and progression [56–58].

Long-chain non-coding RNA (lncRNA) is a kind of RNA molecule characterized by a transcript length exceeding 200 nucleotides, which causes a lack of the capability to encode proteins. Initially, this particular RNA was regarded as "noise" of genome transcription. Since Hotair's functional identification in 2007, the function of lncRNA has gradually become clear. It was determined that around 93% of the transcripts were classified as lncRNAs, which are typically located in both the nucleus and the cytoplasm [59]. Although the transcription level of lncRNA is typically less than that of protein-coding genes, its sequence conservation is generally poor and evolutionary pressure is minimal. Nonetheless, the promoter sequence tends to be relatively conservative. LncRNA exhibits a longer sequence length and a more intricate spatial structure in contrast to small-molecule RNA. Furthermore, the regulatory mechanisms that control its expression are characterized by greater diversity and complexity. RNA capture long seq (CLS) can analyze the genome with greater precision to gain insight into lncRNA [60]. According to the relationship between lncRNA and the coding gene, lncRNA can be distributed into seven classifications: (1) antisense lncRNAs: transcribed from the reverse DNA sequence of protein-coding genes; (2) sense lncRNAs: transcribed from protein-coding genes, lncRNA shares part or all DNA sequence with the protein-coding gene; (3) intraonic lncRNA: derived from the intronic sequence; (4) intergenic lncRNA, derived from the sequence between two genes; (5) overlapping transcribed lncRNA: the coding sequence is lncRNA that spans introns and exons; (6) bidirectional lncRNA: its transcription start site is the same as that of the protein-coding gene on the opposite strand. Transcription start sites are very close (less than 1000 bases), but the direction of transcription is opposite; (7) Others: such as eRNA and circRNA. lncRNA is an important regulator in the human genome, and its function depends on the subcellular location and the molecules that interact with it. lncRNA plays a crucial role in regulating gene expression at numerous levels, like epigenetic, transcriptional, and post-transcriptional levels in the nucleus. Similarly, lncRNA in the cytoplasm is involved in modulating gene expression at the level of translation or post-translation. Post-transcriptional regulation of gene expression has a vital function in various regulatory mechanisms, including but not limited to the development, differentiation, metabolism, X chromosome inactivation, genome imprinting, chromatin modification, transcriptional activation and repression, and nuclear transport. Furthermore, it is closely associated with the onset, progression, and prevention of human diseases. These entities exhibit a high degree of correlation [61]. More and more investigations have revealed that abnormal lncRNA expression is related to the onset, development, recurrence, metastasis, and resistance to chemotherapy of tumors, which can help with diagnosis and prognosis and provide therapeutic targets [62, 63]. For example, the RAS and MYC oncogenes can contribute to tumor incidence and progression through the lncRNA Orilnc1 and DANCR, respectively [64, 65]. The study suggests that lncRNA XIST may facilitate TGF-beta-induced EMT in non-small cell lung cancer through the modulation of the miR-367/141-ZEB2 signaling mechanism [66]. LncRNA RAMS11 has been found to enhance the process of invasion and metastasis in colorectal carcinoma, and its presence is indicative of a poor prognosis [67]. The activation of the STAT3/VEGFA signaling axis by lncRNA PVT1 has been found to enhance angiogenesis in gastric cancer [68]. Bladder cancer cells secrete exosomes containing LncRNA LNMAT2 and upregulate PROX1 expression to promote lymphangiogenesis and lymphatic metastasis [69]. lncRNA is extracellularly released via exosomes. The presence of lncRNA in bodily fluids has the potential to function as a marker for tumor progression and malignancy, as well as to provide therapeutic targets to guide personalized treatment [70]. PCA3 is a lncRNA that exhibits prostate cancer-specific expression. It has been observed to be significantly upregulated in the urine of individuals with prostate cancer and has been employed in the clinical diagnosis of this disease [71]. lncRNA in the plasma extravesicles of individuals with pancreatic ductal adenocarcinoma can distinguish tumors well from non-tumor patients and can also identify surgically resectable stage I/II in patients with pancreatic ductal adenocarcinoma, which has good clinical diagnostic value [72]. The diagnostic efficacy of HULC has been observed to be high in several types of cancers, including non-small cell lung, hepatic, gastric, and pancreatic cancers [73].

Tumor immune escape originates from the immune surveillance theory proposed by Burnet et al. They believe that the body's immune system can examine "non-self" mutant cells and specifically eliminate mutant cells through cellular immune pathways to preserve the stability of the body's internal environment [74]. In certain cases, cancerous cells have the ability to escape the immune system's surveillance through different processes, leading to their rapid proliferation within the body and subsequent formation of tumors, thereby resulting in immune evasion [75]. Tumor immune escape is induced by many factors, including recognition of tumor-specific antibodies as self-antigens, regulation of tumor surface antigens, tumor-induced immune zones, low immunogenicity of tumor cells, and tumor-induced immunosuppression [76]. There are differences in immunogenicity among tumor cells. Tumor cells that exhibit high immunogenicity can be eliminated by causing a potent anti-tumor immune response, whereas tumor cells that display comparatively low immunogenicity can evade immune surveillance and undergo selective proliferation. Following the immune selection process of body against tumors, the immunogenicity of tumors gradually diminishes, thereby escaping the surveillance of body's immune system [77]. The deficient ability to present MHC class I molecules has been identified as a primary factor contributing to immune evasion by tumors. Polycomb repressive complex 2 (PRC2) has the ability to initiate synchronized transcriptional suppression of MHC-I antigen processing mechanisms, which consequently promotes the evasion of immune surveillance by T cells [14]. Recently, the exploration of tumor immunotherapy has been facilitated by the comprehensive investigation of pathway of tumor immune evasion. Recent investigations have indicated an association between the expression of PD-L1 and EMT. Epithelial tumor cells exhibit decreased expression of PD-L1, whereas mesenchymal tumor cells that underwent EMT transformation reveal elevated levels of PD-L1 expression. It can evade immune surveillance with ease [78]. The majority of malignancy immunotherapies, such as immune checkpoint blockade, are designed to address immune evasion by turning the scales towards immune activation. This approach enables the T-cells-mediated eradication of tumor cell [79]. Nevertheless, the effectiveness of immunotherapy is restricted to a minor category of patients, thereby necessitating urgent identification of genomic and molecular determinants that underlie immune evasion [36].

There are currently few reports on the relationship between LINC01592 and tumors. There is only one report on LINC01592 as a prognostic marker for esophageal cancer [80]. As a common transcription factor, E2F6 has been reported in many tumors, especially in glioma [81], liver cancer [82], gastric cancer [83], bladder cancer [84], etc. It is highly expressed in tumor tissues and is inversely proportional to patient prognosis. The autophagy transport receptor (NBR1) is an autophagy receptor for ubiquitinated proteins in selective autophagy and has been shown to direct the selective autophagic degradation of midbody derivatives and peroxisomes [85]. A recent study shows that NBR1 binds to MHC-I through its ubiquitin-binding domain and mediates the degradation of MHC-I molecules in autophagy lysosomes, thereby promoting immune evasion of pancreatic cancer [15].

Of course, we also investigated the upstream regulatory mechanism of LINC01592 in M2-TAMs, which is crucial for elucidating the complete rationale and theoretical basis of the study. This would also enable them to clarify how LINC01592 is secreted by M2-TAMs and delivered to tumor cells via exosomes. JASPAR and UCSC analyses of the region ~ 2 kb upstream of the genome sequence of LINC01592 revealed one predicted HIF-1α binding site in the promoter region (Fig. S12B). Pretreatment with hypoxia or CoCl_2_ for one day, evidently elevated the LINC01592 and HIF-1α levels (Fig. S12C). In contrast, the knockdown of HIF-1α gene under normoxic and hypoxic conditions obviously suppressed the transcription of LINC01592 (Fig. S12D). Subsequent ChIP–qPCR results indicated that HIF-1α directly bound to the same chromatin fragment of the promoter region of the LINC01592 gene (Fig. S12E). To further verify if HIF-1α activated LINC01592 transcription, we cloned the LINC01592 5′UTR fragment containing the wild type (WT) or mutant (MUT) hormone response element (HRE)-binding sequences into the promoter regions of the pGL3-control plasmids. Hypoxia promoted the luciferase intensity in Eca-109 and PECC cells with the WT promoter, while there was no significant difference in Eca-109 and PECC cells containing the MUT promoter. However, knocking down the HIF-1α gene obviously inhibited the luciferase intensity containing the WT promoter (Fig. S12F-G). In conclusion, our data proved that LINC01592 is a direct transcriptional target of HIF-1α.

**Fig. 9:**
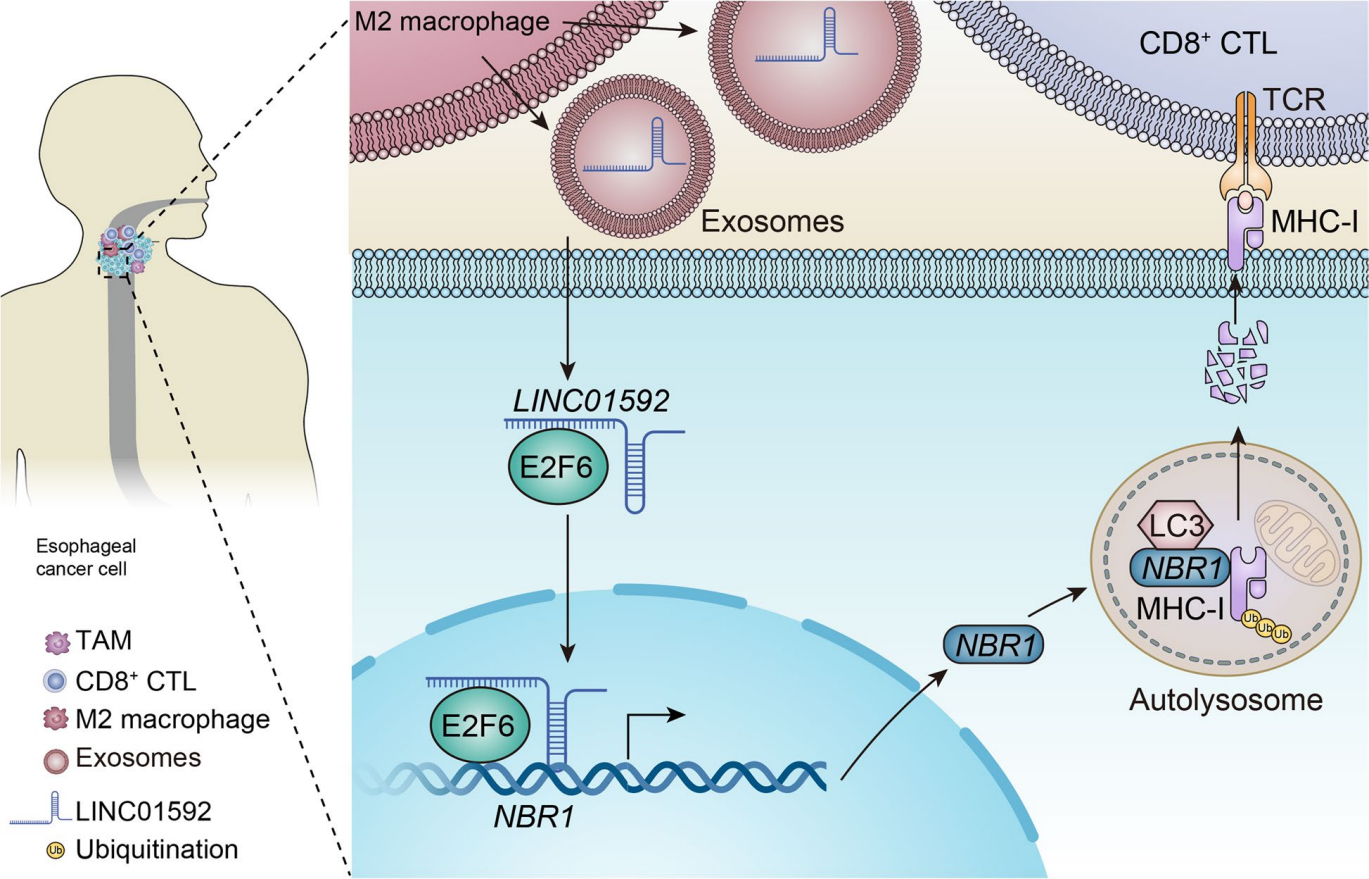
Schematic diagram of the mechanism. T2-TAMs secrete exosomes rich in LINC01592 into tumor cells, and LINC01592 directly binds E2F6 and promotes E2F6 entry into the nucleus; the two synergistically promote the transcriptional level of NBR1. The increased binding of NBR1 to the ubiquitinated protein MHC-I through the ubiquitin domain mediates the increased degradation of MHC-I in autophagolysosomes and the decreased expression of MHC-I on the surface of tumor cells, which in turn leads to tumor cells escaping CD8^+^ CTL's immune attack

In this investigation, it was demonstrated that the origin of LINC01592 from M2-TAM exosomes enhanced evasion of the immune system by the tumor through the activation of E2F6/NBR1/MHC-I signaling pathway. The tumor-promoting effects of LINC01592 were significantly reduced through the disruption of E2F6/NBR1/MHC-I signal axis. Interestingly, the suppression of LINC01592 resulted in an enhancement of MHC-I expression on the tumor cells surface, thereby enhancing the efficacy of CD8^+^ T cell reinfusion (Fig. 9).

### Supplementary Information

**Additional file 1.**

## Data Availability

Not applicable.
