# Supplementary material for: Tumor-associated macrophage-derived exosomes LINC01592 induce the immune escape of esophageal cancer by decreasing MHC-I surface expression
Source: J Exp Clin Cancer Res. 2023 Nov 2;42:289. doi: 10.1186/s13046-023-02871-2 (PMC10621170; doi:10.1186/s13046-023-02871-2)
Supplement: Supplementary file 1 — Additional file 1. [file 13046_2023_2871_MOESM1_ESM.docx]

**Tumor-associated Macrophage-derived Exosomes LINC01592 Induce the Immune Escape of Esophageal Cancer by Decreasing MHC-I Surface Expression**

Xinwei Qiao^1#^, Zaixing Cheng^1#^, Kaming Xue^2^, Cui Xiong^3^, Zhikun Zheng^1^, Xin Jin^4^, Jinsong Li^1^

**Materials and methods**

**Patients and clinical samples**

All fresh EC specimens were obtained from the patients operated at the Wuhan Union Hospital. This study has obtained the approval of the relevant institutional review board of Tongji Medical College of Huazhong University of Science and Technology. All specimens were in quadruplicates, each stored in (i) tissue preservation solution for primary culture, (ii) 4% paraformaldehyde for fixation, (iii) in situ hybridization preservation solution for fixation, and (iv) liquid nitrogen for subsequent experiments. Before collecting the specimens, we obtained "informed written consent" from patients. No patient received radiotherapy or chemotherapy before the surgery. This research was performed according to the International Ethical Guidelines for Biomedical Research Involving Human Subjects issued by the Council for International Organization of Medical Sciences (CIOMS). Details on patient characteristics are provided in Tables S1-2. Before specimen collection, all patients signed informed consent. The study was conducted in accordance with the guidelines in the Declaration of Helsinki and the relevant ethical approvals were obtained. All patients were graded according to the latest diagnosis and treatment guidelines for esophageal cancer.

**Cell culture and treatment**

The human esophageal cancer cell lines Eca-109 were purchased from the Chinese Academy of Science Cell Bank. Het-1A, TE-1 and KYSE-150 cells were purchased from Procell life Science & Technology Co.Ltd. (China). All cells were cultured in RPMI-1640 (PM150110, Procell, China) plus 10% FBS (164210-500, Procell, China) and 1% P/S (PB180120, Procell, China) maintained at 37 °C with 5% CO2. All cell lines were authenticated by short tandem repeat (STR) and verified to be free of mycoplasma contamination. For methods of extraction and culture of primary esophageal cancer cells (PECC), please refer to our previously published articles [1].

**Plasmids, small interfering RNA (siRNA), and transfection**

GeneChem Co. Ltd. (Shanghai, China) synthesized the lentiviral overexpression plasmids for LINC01592, E2F6 and NBR1, as well as the lentiviral knockdown plasmids for LINC01592, E2F6 and NBR1. The sequences of used shRNAs and siRNAs were listed in Table S3. PCR was used to amplify and clone the truncated structure cDNAs of Human LINC01592 into the pECMV-3xFLAG-C expression vector. For transient transfections, Lipofectamine 3000 from TermoFisher Scientifc in the USA was utilized following the guidelines provided by the manufacturer. Subsequently, colonies expressing cells were chosen by applying 2 mg/ml puromycin for stable expression.

**Real-time quantitative RT-PCR (qRT-PCR)**

The total RNA from tissues or cell lines was extracted using Trizol (Takara, Otsu, Japan) according to the manufacturer's instructions. With SYBR Green premix Pro Taq HS qPCR Kit (AG11728, Accurate Biotechnology Co., Ltd, Hunan, China), 1 ug of total RNA was reverse transcribed into cDNA using Evo M-MLV RT Kit. Real-time PCR reactions were conducted using the BioRad system (BioRad , USA). To normalize RT-qPCR results, GAPDH was used as the internal reference , and relative expression was calculated using the 2(-ΔΔCT) method. Primer sequences are listed in Table S5.

**Bioinformatic analysis**

The transcript data of EC samples together with clinical information was downloaded from TCGA (https://cancergenome.nih.gov/). The transcript data of normal brain tissues was downloaded from the Genotype Tissue Expression (GTEx, https://gtexportal.org/home/) [2]. UCSC XENA (https://xenabrowser.net/datapages/) RNAseq data in TPM format of TCGA and GTEx uniformly processed by the Toil process [3]. RNAseq data in TPM (transcripts per million reads) format and log2 transformation for expression comparison between samples. RNAseq data in FPKM (Fregments Per Kilobase per Million) format was converted into TPM (transcripts per million reads) format and log2 conversion was performe [4]. Prognostic data from the following related articles. The results of the independent sample *t*-tests comparing the two groups are expressed as *t*-test values.

**Statistical analysis**

All statistical analyses were conducted with SPSS 23.0 software (SPSS, Inc., Chicago, IL) and GraphPad Prism Software (GraphPad Prism version 8.3.1 for Windows; GraphPad Software, www.graphpad.com). All data were presented as the mean ± sd. Unpaired/paired Student’s t test for two groups or one-way ANOVA + Dunnett’s for more than two groups, which was used to assess statistically significant data. The chi-square test, Pearson’s correlation, and one-way analysis of variance were also performed. Cox regression analysis and Log-rank test were used to determine survival difference and hazard ratio. P-values < 0.05 were considered to be statistically significant.

**Table S1:** Correlation of the expression levels of LINC01592 in EC tissues with clinicopathologic features.

| **Characteristics** | **No. of cases (%)** | **LINC01592** | | **P-value** |
| --- | --- | --- | --- | --- |
|  |  | **Low** | **High** |  |
| **Age (y)** |  | | | |
| ＜50 | 46 (38.98%) | 21 | 25 | 0.909 |
| ≥50 | 72 (61.02%) | 32 | 40 |  |
| **Gender** |  | | | |
| Male | 65 (43.8%) | 30 | 35 | 0.766 |
| Female | 53 (56.2%) | 23 | 30 |  |
| **Tumor size (cm)** |  | | | |
| ＜2 | 76 (64.41%) | 43 | 33 | 0.0006*** |
| ≥2 | 42 (35.59%) | 10 | 32 |  |
| **Mortality** |  | | | |
| Survive | 68 (57.63%) | 38 | 30 | 0.005** |
| die | 50 (42.37%) | 15 | 35 |  |
| **TNM stage** |  | | | |
| I | 20 (16.95%) | 12 | 8 | 0.017* |
| II | 55 (46.61%) | 30 | 25 |  |
| III | 38 (32.20%) | 10 | 28 |  |
| IV | 5 (4.24%) | 1 | 4 |  |
| **T stage** |  | | | |
| T1 | 26 (22.03%) | 20 | 6 | 0.0003*** |
| T2 | 63 (53.39%) | 30 | 33 |  |
| T3 | 21 (17.80%) | 3 | 18 |  |
| T4 | 8 (6.78%) | 0 | 8 |  |
| **Lymph node metastasis** |  | | | |
| N0 | 32 (27.12%) | 23 | 9 | 0.024* |
| N1 | 46 (38.98%) | 20 | 26 |  |
| N2 | 34 (28.81%) | 10 | 24 |  |
| N3 | 6 (5.09%) | 0 | 6 |  |
| **Distant metastasis** |  |  |  |  |
| M0 | 112 (94.92%) | 53 | 59 | 0.011* |
| M1 | 6 (5.08%) | 0 | 6 |  |

*<0.05, **<0.01 and ***<0.01. LINC01592 high expression: score 8-16; low expression: score 0-7.

**Table S2:** Univariate and multivariate for clinicopathological features associated with various prognostic parameters of 118 EC patients by Cox-regression analysis.

| Variables | Univariate analysis | | Multivariate analysis | |
| --- | --- | --- | --- | --- |
|  | HR (95%CI) | P-value | HR (95%CI) | P-value |
| Age (≥50 vs ＜50) | 1.128 (0.594-2.138) | 0.592 | 1.096 (0.643-2.352) | 0.639 |
| Gender ( Male vs Female) | 1.312 (0.976-2.693) | 0.412 | 1.235 (0.851-2.413) | 0.436 |
| Tumor size (≥2 vs ＜2) | 2.468 (1.496-3.752) | 0.0075** | 2.154 (1.362-3.381) | 0.018* |
| TNM stage | 1.964 (1.259-2.937) | 0.023* | 1.729 (1.352-3.149) | 0.045* |
| T stage | 2.753 (1.639-4.216) | 0.0038** | 1.912 (1.254-3.268) | 0.026* |
| Lymph node metastasis | 1.693 (1.285-2.796) | 0.042* | 1.468 (1.136-2.745) | 0.068 |
| Distant metastasis | 1.512 (1.135-2.637) | 0.056 | 1.357 (1.042-2.573) | 0.185 |
| Mortality | 2.037 (1.369-4.253) | 0.015* | 1.852 (1.264-3.736) | 0.035* |

**Supplementary Tables 3:**

| **Gene** | **Primer** | **Sequence(5′-3′)** |
| --- | --- | --- |
| si-1592#1 | forward | GCCTATTGTTATTGGGCAT |
|  | reverse | CGGATAACAATAACCCGTA |
| si-1592#2 | forward | TGAATGAAAGAAAAGCAGTAT |
|  | reverse | ACTTACTTTCTTTTCGTCATA |
| si-E2F6#1 | forward | GACCTCGTTTTGATGTATCGCTGG |
|  | reverse | CTGGAGCAAAACTACATAGCGACC |
| si-E2F6#2 | forward | ATCATCACTGGACTGGTGGGTCGTACTG |
|  | reverse | TAGTAGTGACCTGACCACCCAGCATGAC |
| si-NBR1#1 | forward | AGATGGCAGTTAAACAGGGAAAC |
|  | reverse | GTGGGGCTTCATCAACGACA |
| si-NBR1#2 | forward | CAGTGCAGTCGTTTCCACTTG |
|  | reverse | AGGTAGCTTGTGAACCAGTCT |
| sh-1592 | TACTCAACCCCAACTCAACCTCAACTCTCAGTACGGCAGAGAGACGGAGA | |

**Supplementary Tables 4:**

| Name | Description |
| --- | --- |
| Anti-E2F6 antibody | ab289963 (Abcam, Cambridge, USA) |
| Anti-NBR1 antibody | ab55474 (Abcam, Cambridge, USA) |
| Anti-MHC-I antibody | Cat. No. sc-32235 (Santa Cruz, Texas, USA) |
| Anti-CD9 antibody | ab236630 (Abcam, Cambridge, USA) |
| Anti-CD63 antibody | ab134045 (Abcam, Cambridge, USA) |
| Anti-CD81 antibody | ab79559 (Abcam, Cambridge, USA) |
| Anti-HSP70 antibody | ab2787 (Abcam, Cambridge, USA) |
| Anti-TSG101 antibody | ab125011 (Abcam, Cambridge, USA) |
| Anti-GAPDH antibody | #5174 (Cell Signaling Technology, Beverly, MA, USA) |
| Anti-Calnexin antibody | 10427-2-AP (Proteintech, Wuhan, China ) |
| Anti-Alpha Tubulin antibody | 66031-1-lg (Proteintech, Wuhan, China ) |
| Anti-Lamin B1 antibody | #13435 (Cell Signaling Technology, Beverly, MA, USA) |

**Sequences of probes for LINC01592 RNA FISH**

5’-DIG-GGCCCTAAAATAGCTGCTCATTTGTAGCACTCAGACGAAGCTGAAATTAT-DIG-3’

**Supplementary Tables 5:**

| **Gene** | **Primer** | **Sequence(5′-3′)** |
| --- | --- | --- |
| LINC01592 | forward | AGGGCTCAGTAGATTTGCCC |
|  | reverse | CACCTA ACGGAAATGTCGGC |
| E2F6 | forward | TCCATGAACAGATCGTCATTGC |
|  | reverse | TCCGTTGGTGCTCCTTATGTG |
| NBR1 | forward | AGGAGCAAAACGACTAGCTGC |
|  | reverse | TCTGGGGTCTTCATGTCTGAT |
| MHC-I | forward | CCATGAGGTATTTGTGGACCG |
|  | reverse | TCTCGGACTCTCGTCGTCG |
| β-actin | forward | ATTGCCGACAGGATGCAGAA |
|  | reverse | GCTGATCCACATCTGCTGGAA |
| GAPDH | forward | GAGTCAACGGATTTGGTCGT |
|  | reverse | TTGATTTTGGAGGGATCTCG |

**Supplementary Tables 6:**

| **Gene** | **Primer** | **Sequence(5′-3′)** |
| --- | --- | --- |
| ChIP-NBR1 promoter 1-1 | forward | GCGGCTTATTACGTCACATTAATTGCTGTACC |
|  | reverse | CGCCGAATAATGCAGTGTAATTAACGACATGG |
| ChIP-NBR1 promoter 1-1 | forward | GAGTGTTGTTATGTTCTCCTATCTTGAGAGCA |
|  | reverse | CTCACAACAATACAAGAGGATAGAACTCTCGT |
| ChIP-NBR1 promoter 2-1 | forward | ACCTACGGCTGCGAAACACAATTACAATATCC |
|  | reverse | TGGATGCCGACGCTTTGTGTTAATGTTATAGG |
| ChIP-NBR1 promoter 2-2 | forward | TAGAATTATTCTCATAATTCTTATTCTCAT |
|  | reverse | ATCTTAATAAGAGTATTAAGAATAAGAGTA |

**Supplementary figure legends**

**
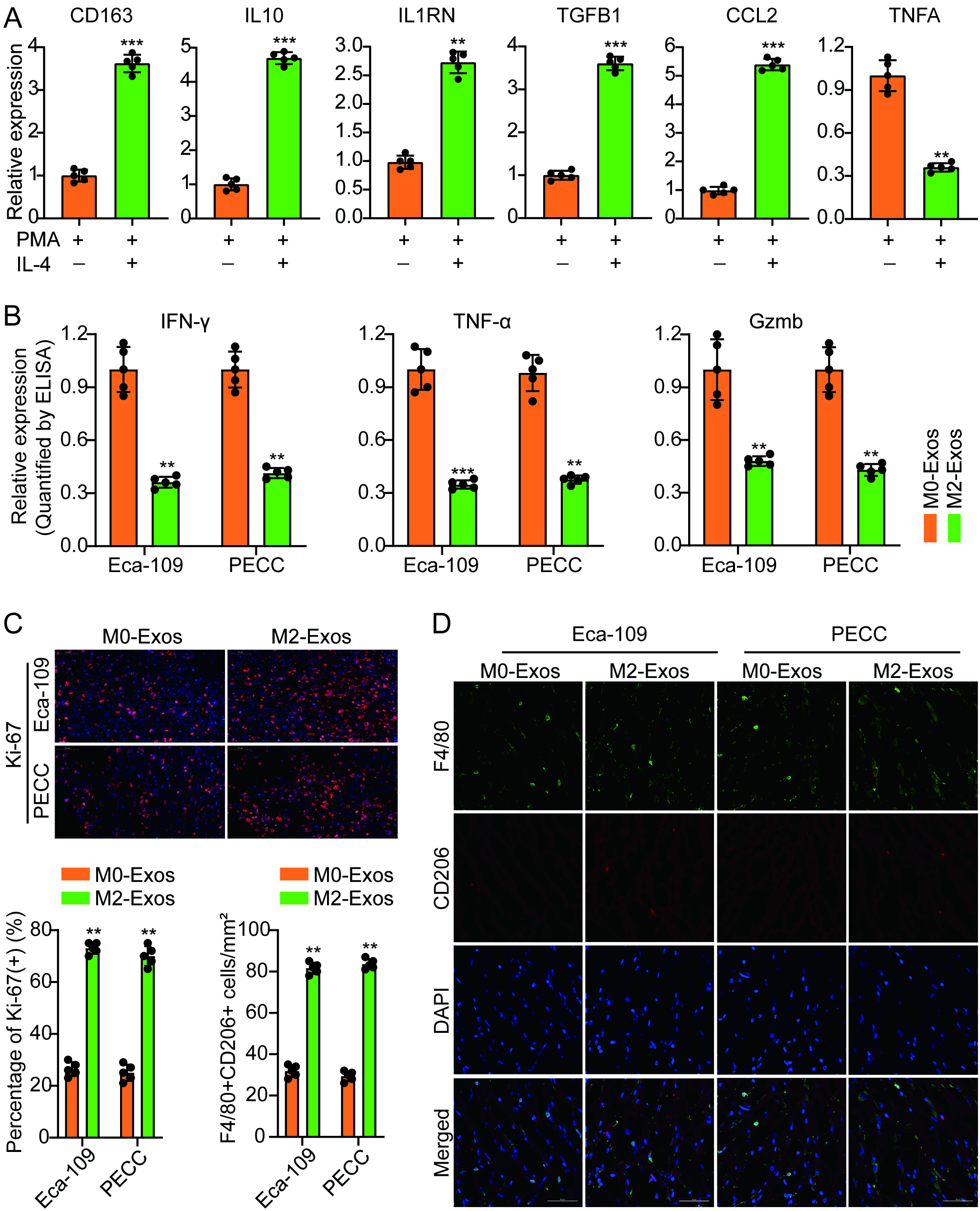
**

**Figure S1**

**A.** The expression levels of CD163, IL10, IL1RN, TGFB1, CCL2 and TNFA were detected by PCR under different processing conditions.

**B.** The expression levels of IFN-γ, TNF-α and Gzmb secretion were measured by ELISA under different processing conditions.

**C.** Typical IF pictures and histogram of Ki-67 under different processing conditions.

**D.** Typical IF pictures and histogram of F4/80 and CD206 under different processing conditions.

The means ± SDs are provided (n=5). **P < 0.01 and ***P < 0.001 according to two-tailed Student t tests or one-way ANOVA followed by Dunnett tests for multiple comparisons.

**
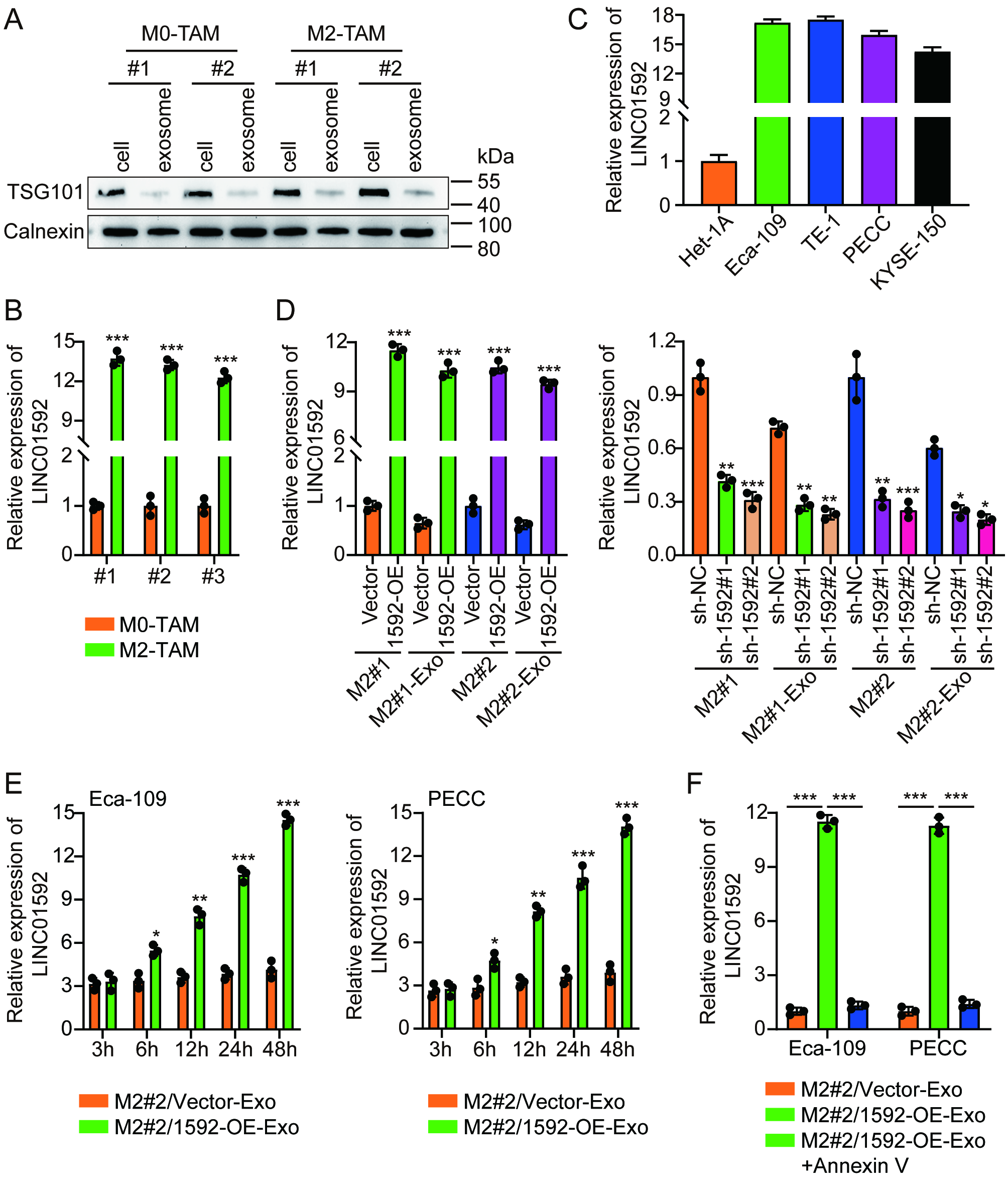
**

**Figure S2**

**A.** The expression levels of TSG101 and Calnexin were measured by PCR under different processing conditions.

**B.** Measuring the levels of LINC01592 in two pairs of M0-TAM and M2-TAM.

**C.** The expression levels of LINC01592 were measured in Het-1A, three EC cell lines (Eca-109, TE-1 and KYSE-150) and primary EC cells (PECC) by PCR.

**D.** The expression levels of exosomal LINC01592 were measured after overexpression or knockdown of LINC01592 in M2-TAM by PCR.

**E.** The expression levels of LINC01592 were measured in Eca-109 and PECC incubated with exosomes derived from M2/Vector and M2/1592-OE.

**F.** The expression levels of LINC01592 were measured in Eca-109 and PECC incubated with exosomes derived from M2/Vector, M2/1592-OE and M2/1592-OE + Annexin V.

The means ± SDs are provided (n=3). *P < 0.05, **P < 0.01 and ***P < 0.001 according to two-tailed Student t tests or one-way ANOVA followed by Dunnett tests for multiple comparisons.


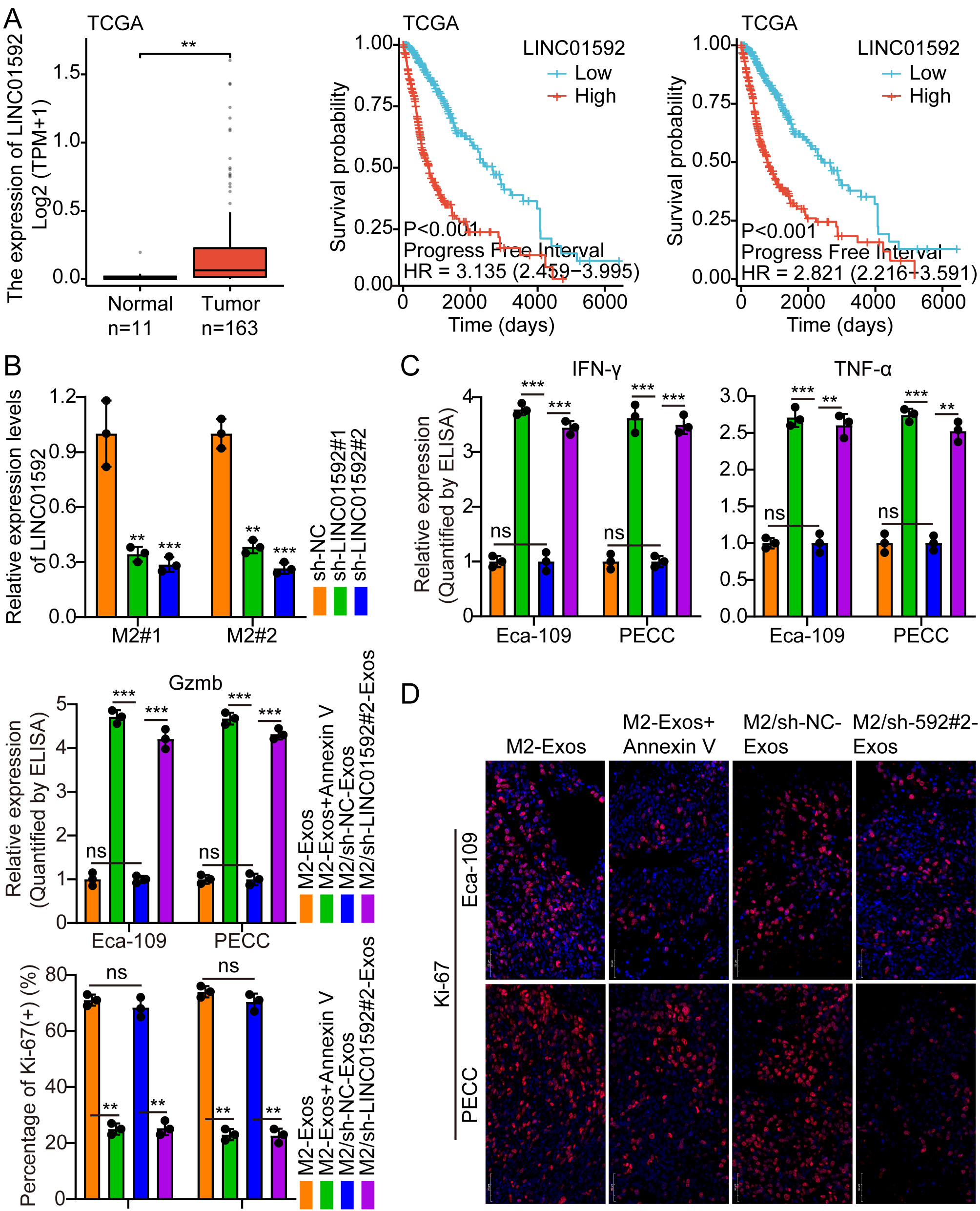


**Figure S3**

**A.** The results of TCGA database indicated that LINC01592 was highly expressed in EC, and the expression levels were inversely proportional to the prognosis of patients**.**

**B.** Validation of knockdown efficiency of LINC01592 by PCR.

**C.** The expression levels of IFN-γ, TNF-α and Gzmb secretion were measured by ELISA under different processing conditions.

**D.** Typical IF pictures and histogram of Ki-67 under different processing conditions.

The means ± SDs are provided (n=3). **P < 0.01 and ***P < 0.001 according to two-tailed Student t tests or one-way ANOVA followed by Dunnett tests for multiple comparisons. ns, no significant difference.


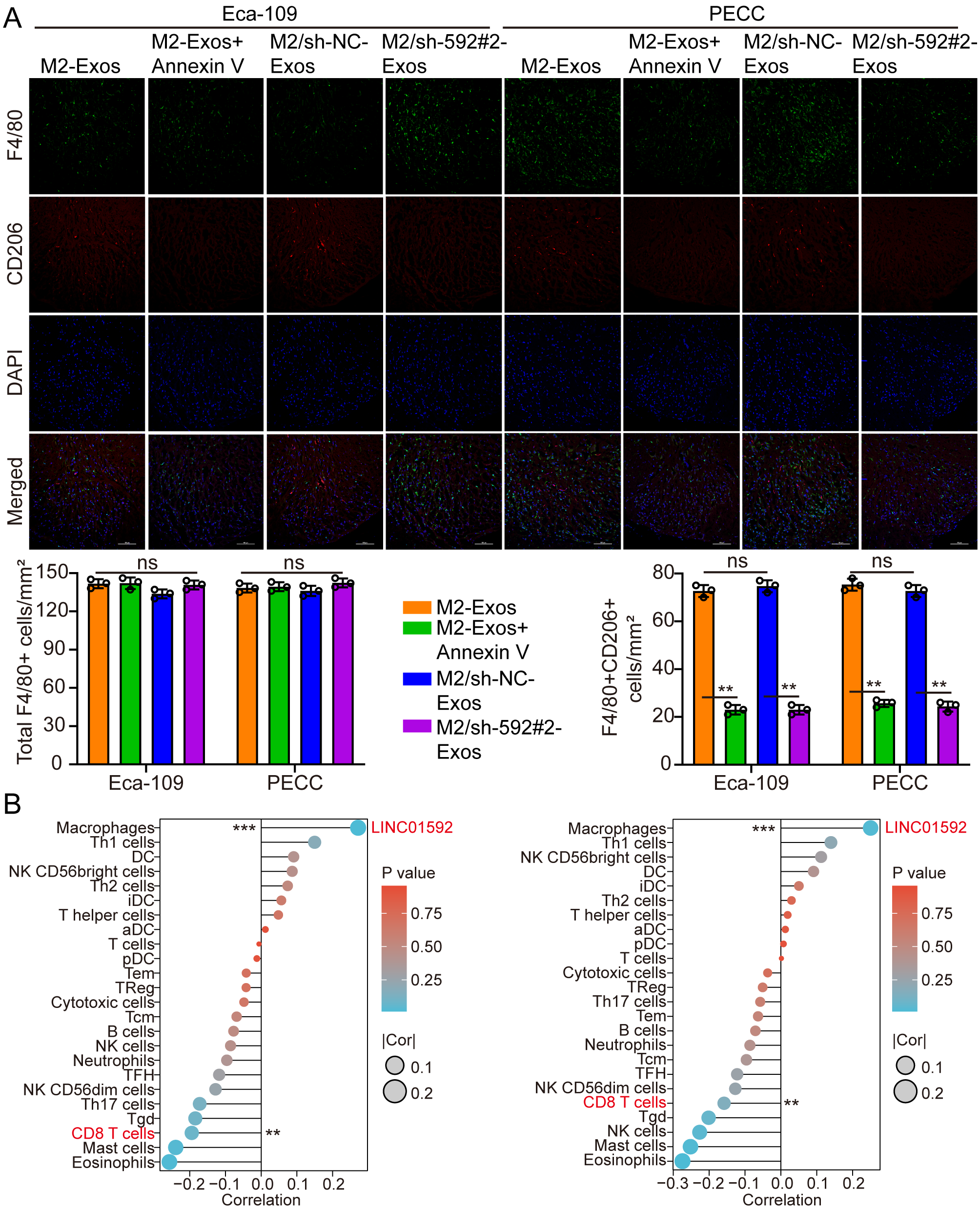


**Figure S4**

**A.** Typical IF pictures and histogram of F4/80 and CD206 under different processing conditions.

**B.** TCGA database analysis demonstrated that the expression levels of LINC01592 were inversely proportional to the degree of infiltration of CD8^+^ T cells.

The means ± SDs are provided (n=3). **P < 0.01 and ***P < 0.001 according to two-tailed Student t tests or one-way ANOVA followed by Dunnett tests for multiple comparisons. ns, no significant difference.


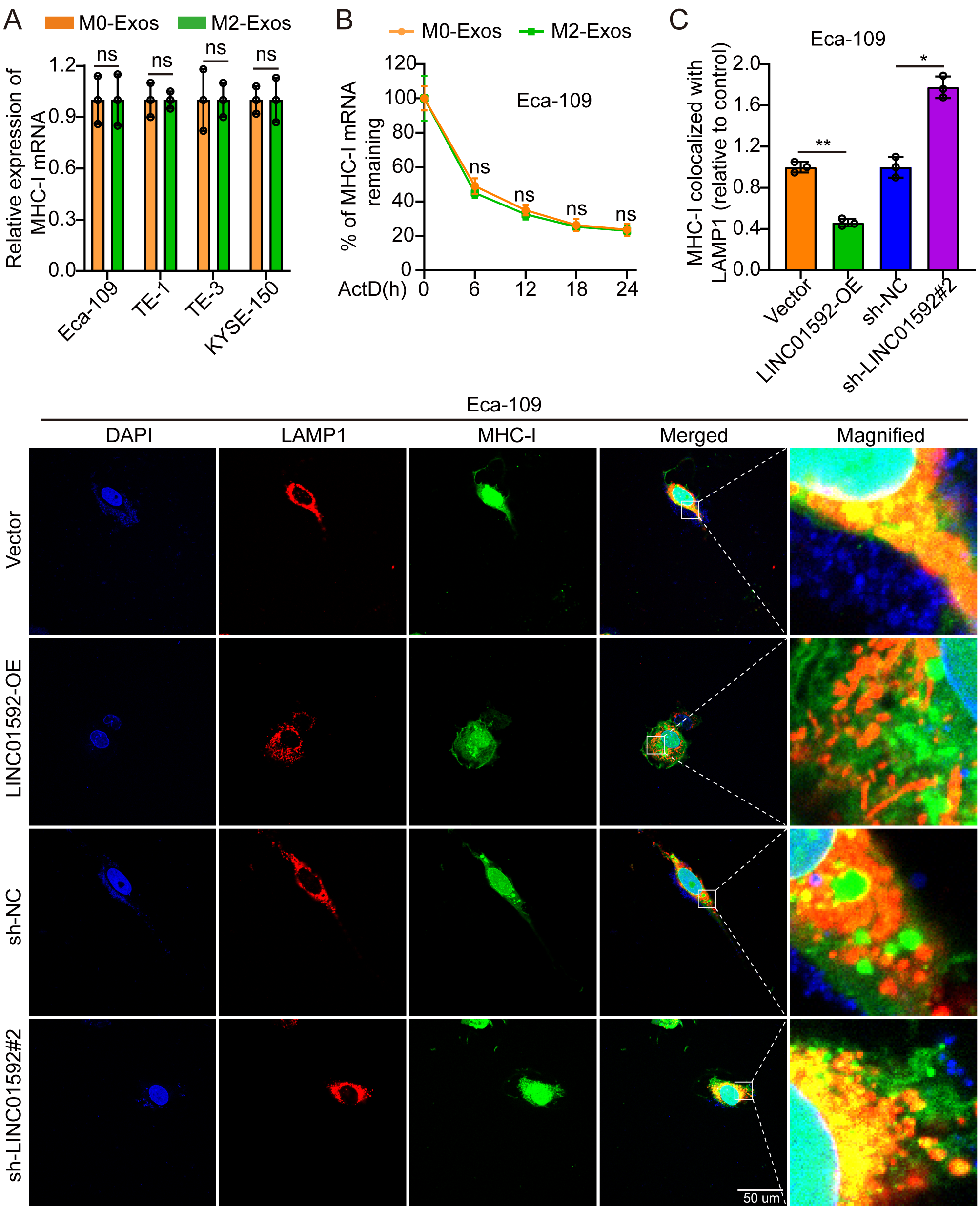


**Figure S5**

**A.** The expression levels of MHC-I were measured by PCR under different processing conditions.

**B.** Typical MHC-I mRNA degradation curve under different processing conditions.

**C.** Representative confocal images showing the expression levels of MHC-I in lysosomes of Eca-109 cells treated with Vector, LINC01592-OE, sh-NC and sh- LINC01592#2. Red represents LAMP1; green represents MHC-I; yellow represents the fusion of red and green.

The means ± SDs are provided (n=3). *P < 0.05 and **P < 0.01 according to two-tailed Student t tests or one-way ANOVA followed by Dunnett tests for multiple comparisons. ns, no significant difference.


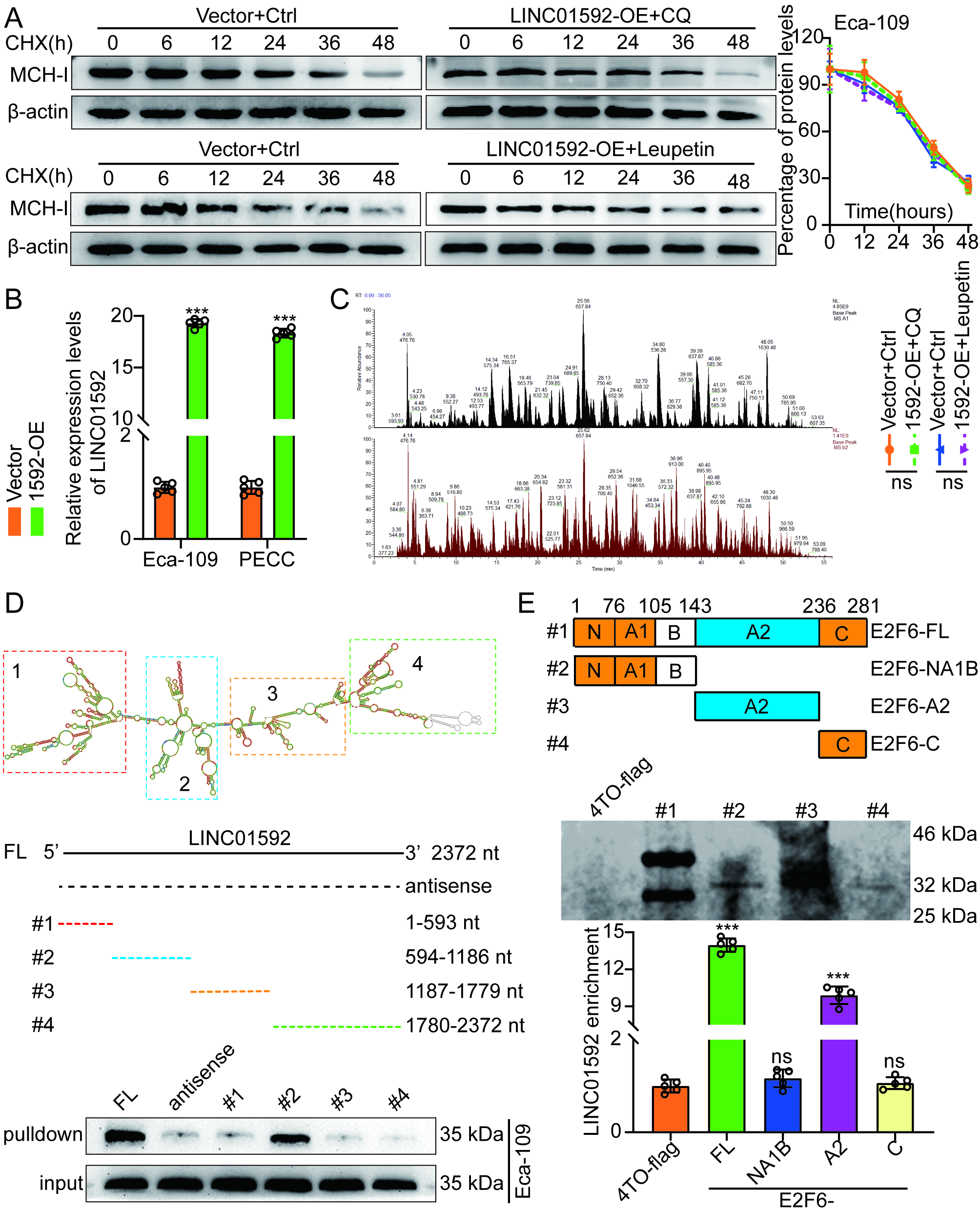


**Figure S6**

**A.** Eca-109 cells expressing either Vector+Ctrl or LINC01592-OE+CQ and expressing either Vector+Ctrl or LINC01592-OE+Leupetin were treated with CHX for corresponding time. Cell lysates were measured by WB with corresponding antibodies. Using Image J to analyze the protein band intensity. Right: Typical MHC-I protein degradation curve.

**B.** Validation of overexpression efficiency of LINC01592 by PCR.

**C.** The mass spectrometry data.

**D.** Immunoblotting detection of the E2F6 proteins in Eca-109 cells as extracted by transcribed biotinylated RNAs in vitro of different constructs of LINC01592 or its antisense sequences (negative control).

**E.** RIP assays were conducted with anti-Flag antibodies in HEK293T cells transfected with Flag-tagged E2F6 vector or its deletion mutants (4TO-Flag vector used as negative control). PCR was used to detect the enrichment of LINC01592. Western blotting was used to measure the expression levels of Flag-tagged E2F6 or its deletion mutants.

The means ± SDs are provided (n=5). ***P < 0.001 according to two-tailed Student t tests or one-way ANOVA followed by Dunnett tests for multiple comparisons. ns, no significant difference.


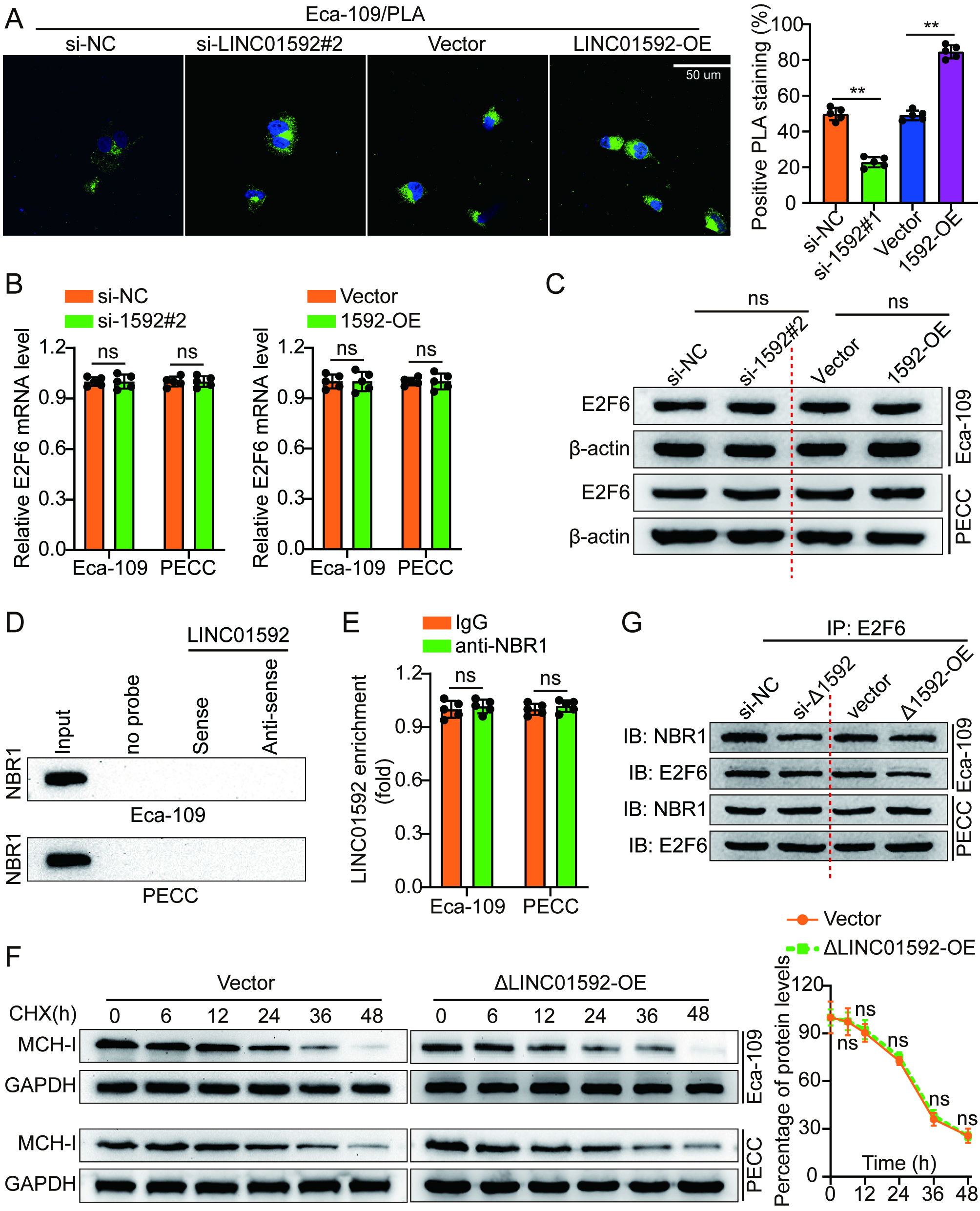


**Figure S7**

**A.** PLA on Eca-109 cells measured the interaction between E2F6 and NBR1 in the LINC01592-knockdown, LINC01592-overexpressing, and corresponding control groups. Positive PLA signal showed that the E2F6-NBR1 complexes were clustered in red and the nuclei we counterstained in blue. Right: Typical histogram.

**B.** The expression levels of E2F6 mRNA were measured by PCR under different conditions.

**C.** The expression levels of E2F6 protein were measured by WB under different conditions.

**D.** WB analysis of the proteins obtained from the LINC01592 pull-down assay with anti-NBR1 antibodies.

**E.** RIP assays with anti-NBR1 antibodies showed that NBR1 did not interact with LINC01592 in Eca-109 and PECC.

**F.** Eca-109 and PECC cells expressing either Vector or ΔLINC01592-OE were treated with CHX for corresponding time. Cell lysates were measured by WB with corresponding antibodies. Using Image J to analyze the protein band intensity. Right: Typical MHC-I protein degradation curve.

**G.** Co-IP assays were conducted to determine the E2F6-NBR1 interaction in the LINC01592 knockdown, LINC01592 overexpressing, and control groups.

The means ± SDs are provided (n=5). **P < 0.01 according to two-tailed Student t tests or one-way ANOVA followed by Dunnett tests for multiple comparisons. ns, no significant difference.


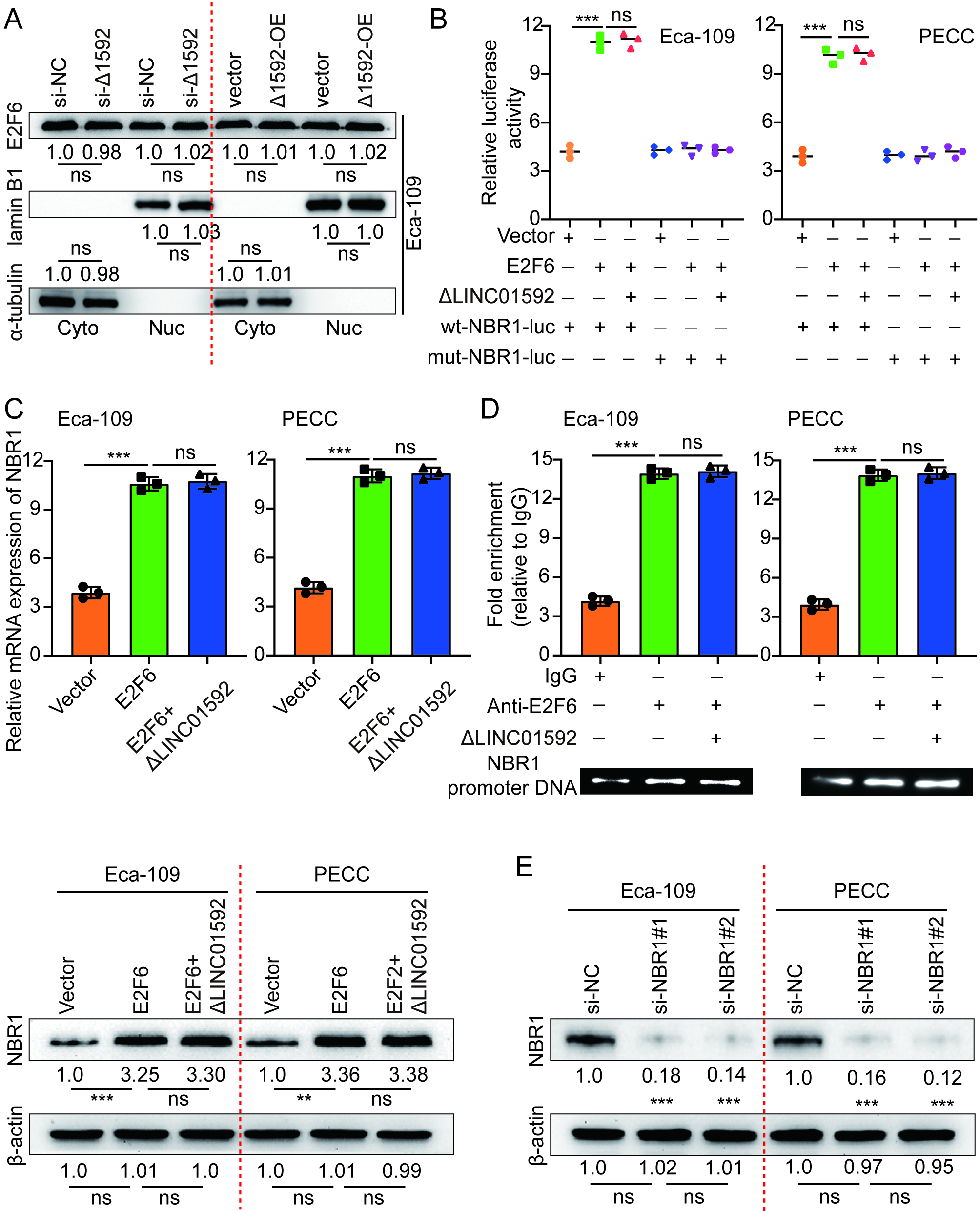


**Figure S8**

**A.** Nuclear and cytosolic lysates were separated from Eca-109 with knockdown or overexpression of ΔLINC01592, followed by WB analysis with corresponding antibodies.

**B.** Luciferase activity was assayed in Eca-109 and PECC cells transfected with luciferase vectors (wild type or mutant type) and meantime co-transfected with expression plasmids (empty vectors, E2F6 expression plasmids, or ΔLINC01592 expression plasmids).

**C.** The levels of NBR1 under ectopic expression of E2F6 or ΔLINC01592 were measured by PCR and WB.

**D.** ChIP experiments of E2F6 (IgG as an internal control) were conducted, and the co-precipitated DNA was subjected to PCR amplification with primers specific to NBR1 promoter region.

**E.** Validation of knockdown efficiency of NBR1 between Eca-109 and PECC by WB.

The means ± SDs are provided (n=3). **P < 0.01 and ***P < 0.001 according to two-tailed Student t tests or one-way ANOVA followed by Dunnett tests for multiple comparisons. ns, no significant difference.


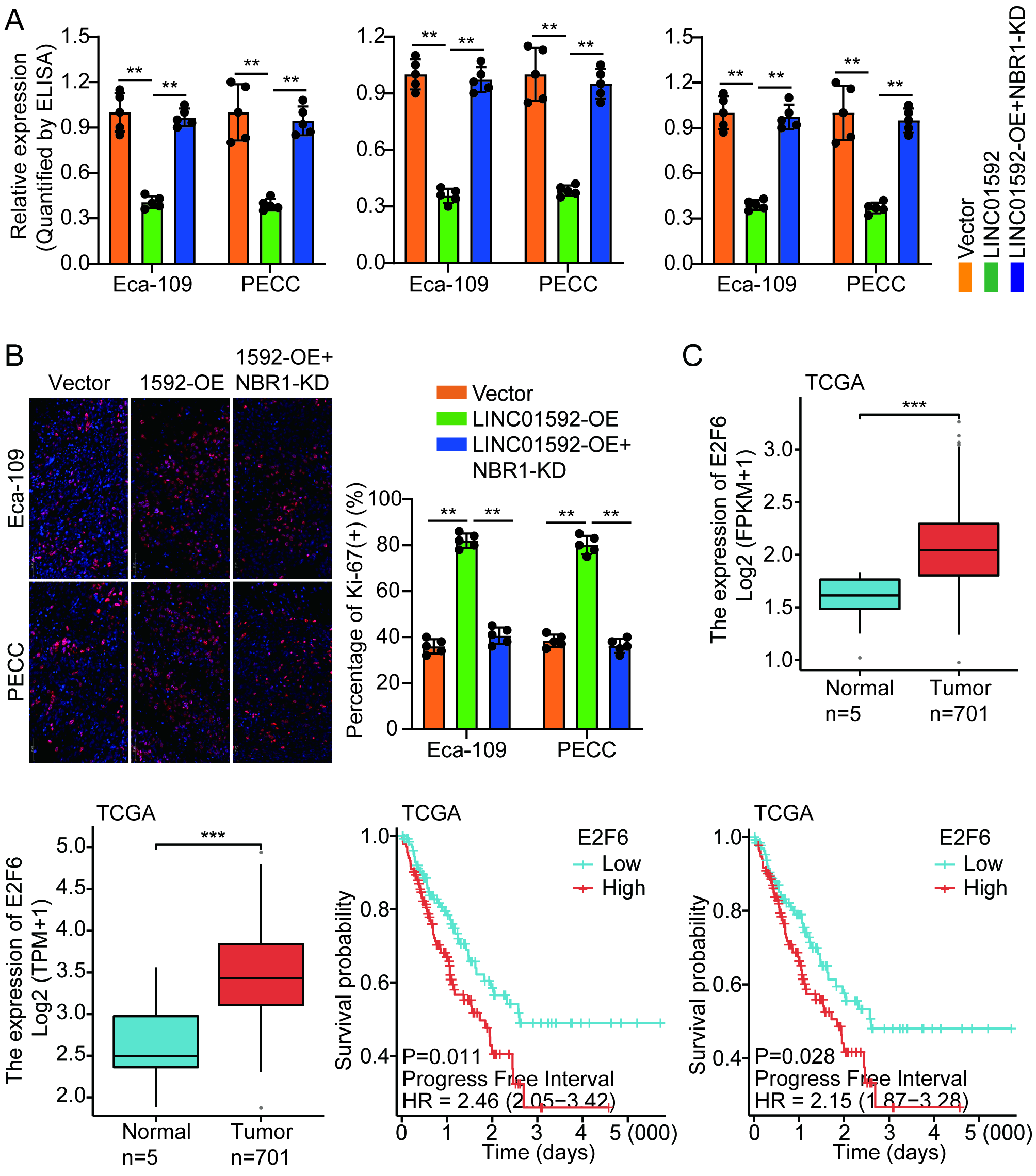


**Figure S9**

**A.** The expression levels of IFN-γ, TNF-α and Gzmb secretion were measured by ELISA under different processing conditions.

**B.** Typical IF pictures and histogram of Ki-67 under different processing conditions.

**C.** The results of TCGA database indicated that E2F6 was highly expressed in EC, and the expression levels were inversely proportional to the prognosis of patients**.**

The means ± SDs are provided (n=5). **P < 0.01 and ***P < 0.001 according to two-tailed Student t tests or one-way ANOVA followed by Dunnett tests for multiple comparisons.


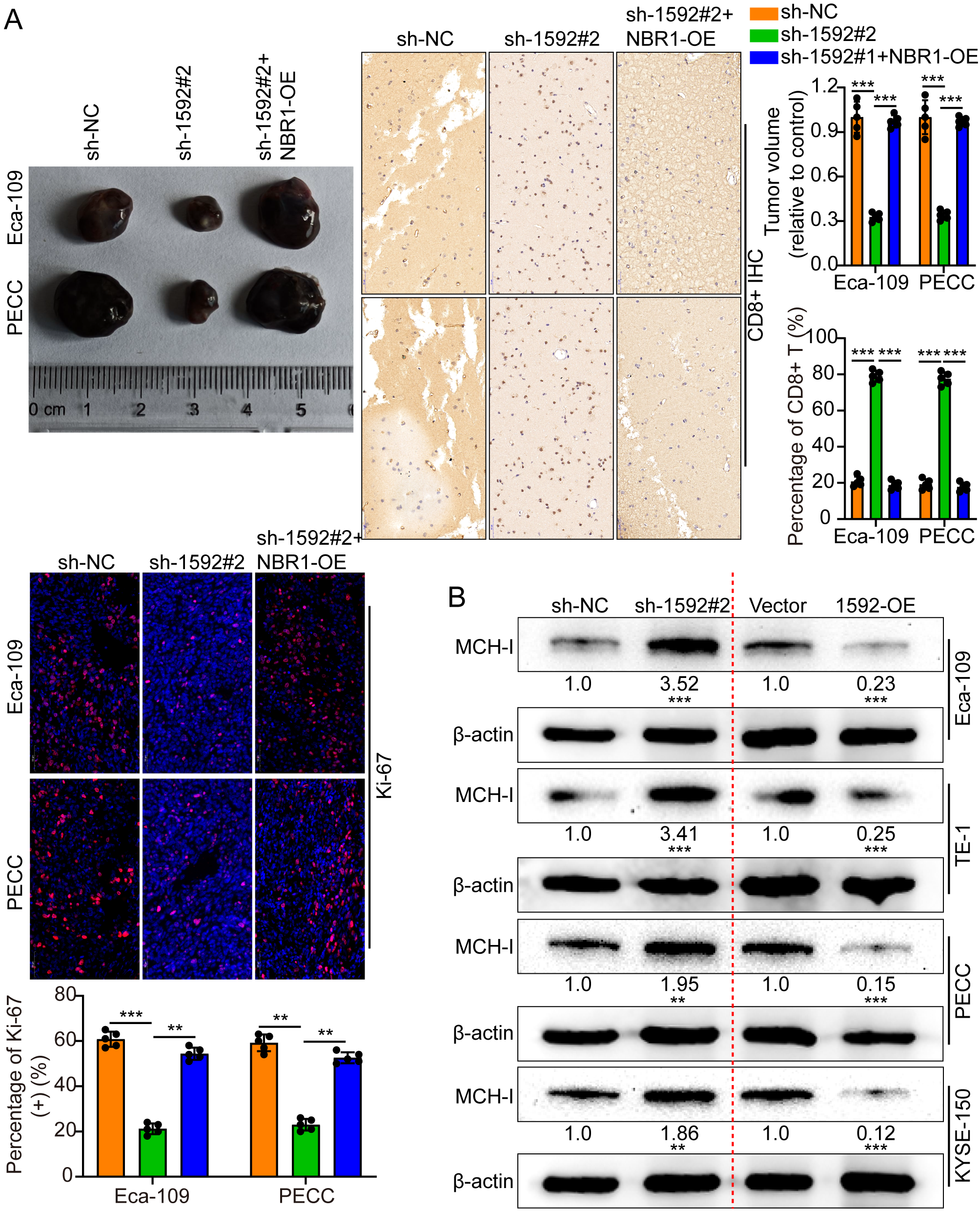


**Figure S10**

**A.** Typical pictures of subcutaneous transplanted tumors, Ki-67 IF pictures and CD8^+^ IHC pictures in different groups. Scale bar, 50um.

**B.** The expression levels of MHC-I protein were measured by WB between sh-NC, sh-LINC01592#2, Vector and LINC01592-OE between Eca-109, TE-1, PECC and KYSE-150.

The means ± SDs are provided (n=5). **P < 0.01 and ***P < 0.001 according to two-tailed Student t tests or one-way ANOVA followed by Dunnett tests for multiple comparisons.


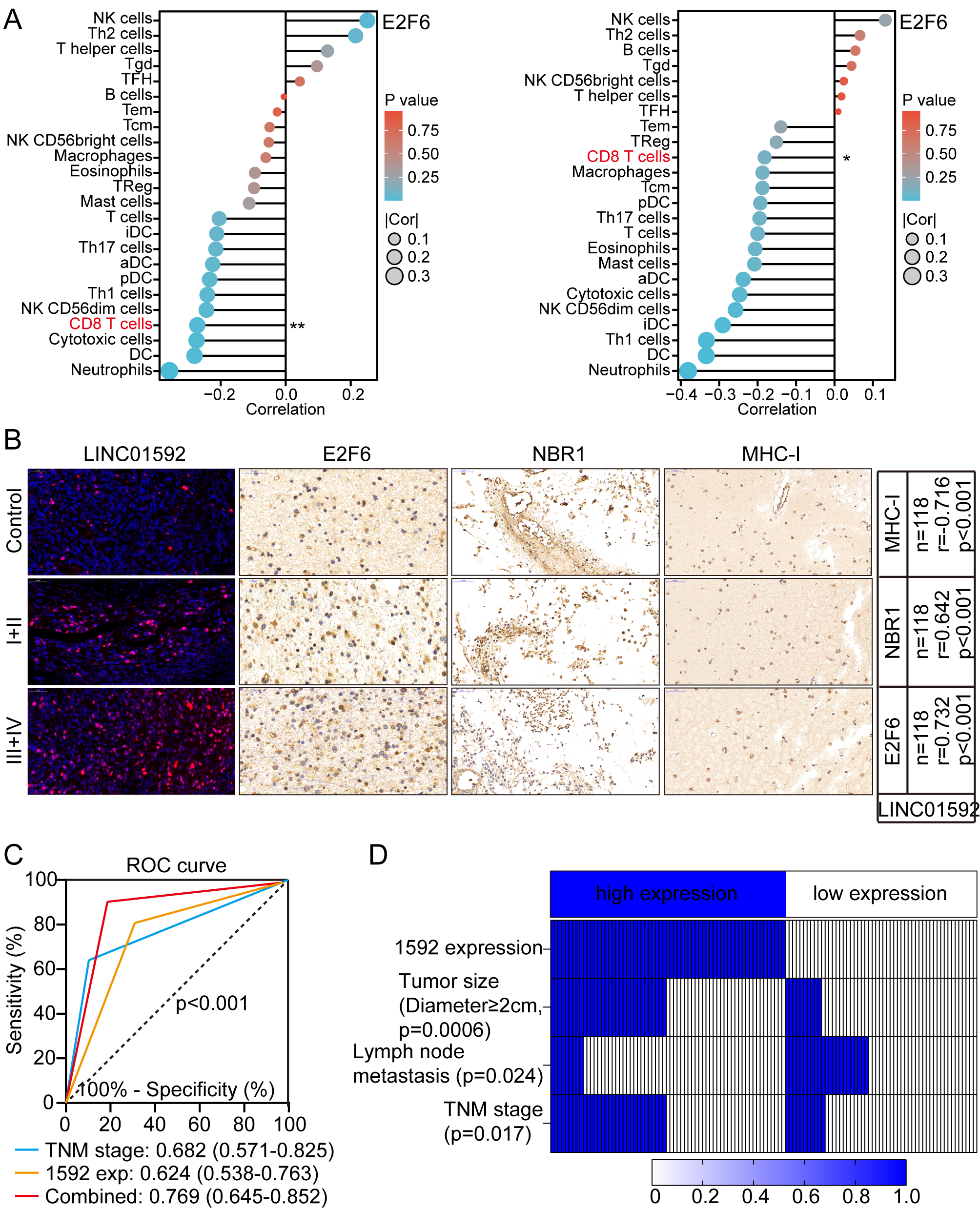


**Figure S11**

**A.** TCGA database analysis indicated that the expression levels of E2F6 were inversely proportional to the degree of infiltration of CD8^+^ T cells.

**B.** Spearman correlation analysis between LINC01592 expression levels and E2F6, NBR1 and MHC-I expression levels in EC. Pearson’s correlation coefficient (r) and P-value as the picture showed; P-value was from Spearman’s test. Scale bar represents 50 μm.

**C.** ROC analysis of LINC01592 expression, TNM stage and the combination model in predicting clinical outcome.

**D.** The heatmap illustrated the association of different clinical characters with LINC01592 high and low-expression tumors.


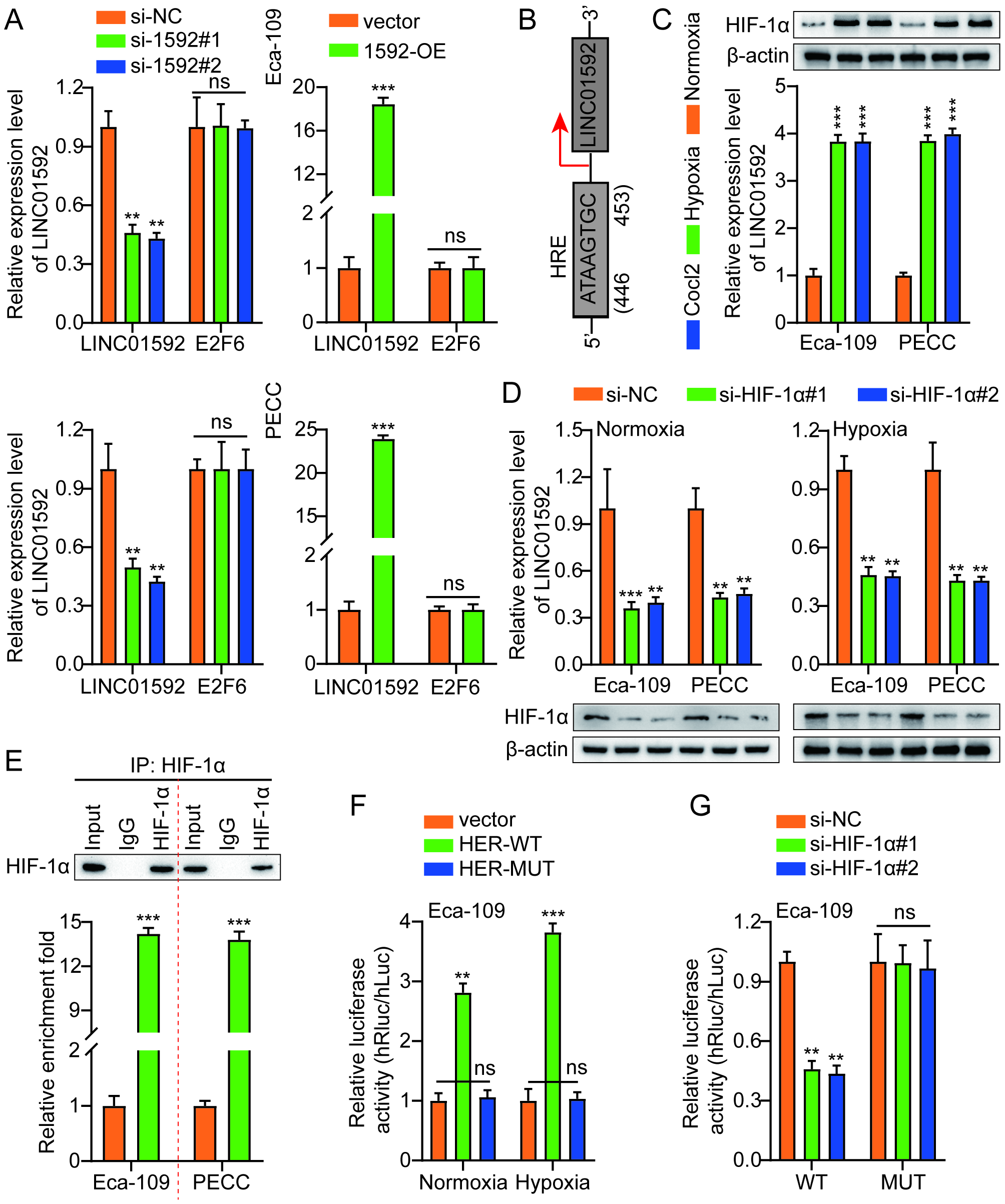


**Figure S12：LINC01592 is induced by hypoxia and can be directly transactivated by HIF-1α.**

1. LINC01592 was knocked down and overexpressed respectively, and the expression levels of E2F6 were detected by qRT-PCR.
2. Schematic illustration of the putative HIF-1α binding site in LINC01592 gene promoter.
3. LINC01592 expression levels (below) and HIF-1α protein (above) in Eca-109 and PECC cells were measured after treated with normoxia, hypoxia or CoCl2 (100 μM) about one day via qRT-PCR and WB.
4. The levels of LINC01592 were measured by qRT-PCR in Eca-109 and PECC cells after HIF-1α knockdown under normoxia or hypoxia condition.
5. ChIP assays with anti-HIF-1α antibodies were performed to measure the binding between HIF-1α and the HRE in LINC01592 promoter in Eca-109 and PECC cells.
6. Eca-109 cells were transfected with pGL3-based vectors containing LINC01592 wide-type promoter (HRE-WT) or mutant-type promoter (HRE-MUT), and further treated with normoxia or hypoxia for two days. Then the firefly luciferase activities were measured and normalized by renilla activities.
7. Eca-109 cells were transfected with pGL3-based vectors containing LINC01592 HRE-WT or HRE-MUT, which were further treated with si-NC, si-HIF-1α for two days. Then the firefly luciferase activities were measured and normalized by renilla activities. Data presented as mean ± sd (n = 3). NS: no significance; **<0.01 and ***<0.001.

**
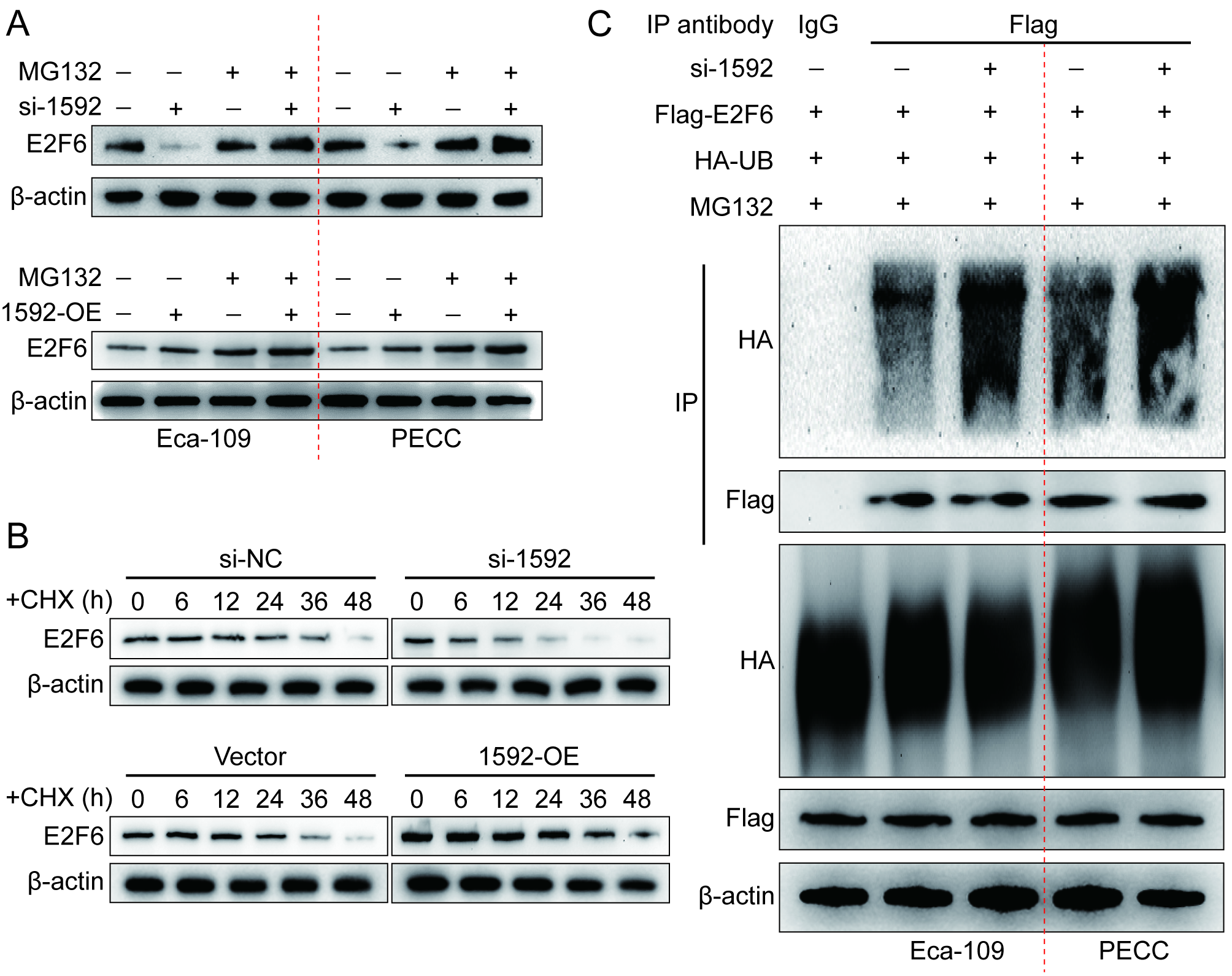
**

**Figure S13：**

**A-B.** Eca-109 and PECC cells expressing either si-NC or si-1592 and expressing either vector or 1592-OE were treated with MG-132 or CHX for corresponding time. Cell lysates were measured by WB with corresponding antibodies.

1. In vitro ubiquitination assays of cells transfected with si-1592 in Eca-109 and PECC cells. All cells were simultaneously co-transfected with Flag-E2F6 and HA-UB plasmids, two days later, all cells were treated with MG-132 for 8 h. Cell lysates were immunoprecipitated with anti-flag antibodies, and then immunoblotted with anti-HA or anti-flag antibodies**.**

**References**

1. Zheng Z, Zhang B, Yu H, Li S, Song N, Jin X, Li J: UBE3A activates the NOTCH pathway and promotes esophageal cancer progression by degradation of ZNF185. *Int J Biol Sci* 2021, 17:3024-3035.

2. Consortium GT: The Genotype-Tissue Expression (GTEx) project. *Nat Genet* 2013, 45:580-585.

3. Vivian J, Rao AA, Nothaft FA, Ketchum C, Armstrong J, Novak A, Pfeil J, Narkizian J, Deran AD, Musselman-Brown A, et al: Toil enables reproducible, open source, big biomedical data analyses. *Nat Biotechnol* 2017, 35:314-316.

4. Liu J, Lichtenberg T, Hoadley KA, Poisson LM, Lazar AJ, Cherniack AD, Kovatich AJ, Benz CC, Levine DA, Lee AV, et al: An Integrated TCGA Pan-Cancer Clinical Data Resource to Drive High-Quality Survival Outcome Analytics. *Cell* 2018, 173:400-416 e411.
